# Towards a characterization of human spatial exploration behavior

**DOI:** 10.3758/s13428-024-02581-3

**Published:** 2025-01-22

**Authors:** Valentin Baumann, Johannes Dambacher, Marit F. L. Ruitenberg, Judith Schomaker, Kerstin Krauel

**Affiliations:** 1 Department of Child and Adolescent Psychiatry and Psychotherapy, University of Magdeburg, Leipziger Strasse 44, 39120 Magdeburg, Germany; 2 Faculty of Computer Science, University of Magdeburg, Leiden, Germany; 3 Institute of Psychology, Department of Health, Medical and Neuropsychology, Leiden University, Leiden, The Netherlands; 4 Leiden Institute for Brain and Cognition, Leiden, The Netherlands; 5 Center for Behavioral Brain Sciences, Magdeburg, Germany

**Keywords:** Spatial exploration, Exploratory behavior, Virtual environment, Novelty seeking, Human exploration

## Abstract

Spatial exploration is a complex behavior that can be used to gain information about developmental processes, personality traits, or mental disorders. Typically, this is done by analyzing movement throughout an unknown environment. However, in human research, until now there has been no overview on how to analyze movement trajectories with regard to exploration. In the current paper, we provide a discussion of the most common movement measures currently used in human research on spatial exploration, and suggest new indices to capture the efficiency of exploration. We additionally analyzed a large dataset (*n* = 409) of human participants exploring a novel virtual environment to investigate whether movement measures could be assigned to meaningful higher-order components. Hierarchical clustering of the different measures revealed three different components of exploration (exploratory behavior, spatial shape, and exploration efficiency) that in part replicate components of spatial exploratory behavior identified in animal studies. A validation of our analysis on a second dataset (*n* = 102) indicated that two of these clusters are stable across different contexts as well as participant samples. For the exploration efficiency cluster, our validation showed that it can be further differentiated into a goal-directed versus a general, area-directed component. By also sharing data and code for our analyses, our results provide much-needed tools for the systematic analysis of human spatial exploration behavior.

**Supplementary Information:**

The online version contains supplementary material available at 10.3758/s13428-024-02581-3.

## Introduction

Whenever humans find themselves in an unknown environment, they use exploration behavior to rapidly acquire information about the new location (Meyer, 1998). Exploration relies on a multitude of cognitive processes, which among others entail memory, motivation, and executive processes such as goal selection and action planning (Düzel et al., 2010; Gottlieb et al., 2013; Johnson et al., 2012; Petzke & Schomaker, 2022; Wolbers & Hegarty, 2010). Measures of exploration behavior have been shown to be sensitive to developmental changes both in childhood and in aging (Henderson et al., 1982; Mata et al., 2013; Schulz et al., 2019; Thurman & Corbetta, 2017), as well as to gender differences (Gagnon et al., 2016; Henderson et al., 1982; Munion et al., 2019). Several studies also observed that exploration reflects individual differences in personality traits such as extraversion (Ai et al., 2019; Alessandretti, Lehmann, et al., 2018) or novelty seeking (Minassian et al., 2022). It may even aid in understanding disorders like autism (Fornasari et al., 2013; Pierce & Courchesne, 2001), schizophrenia (Perry et al., 2010), bipolar disorder (Henry et al., 2010; Young et al., 2007), and dementia (Batrancourt et al., 2019; Kearns et al., 2010, 2011).

In human studies, exploration behavior is typically measured using decision-making tasks, such as a multi-armed bandit task in which players need to explore several options in order to maximize their gains (Brändle et al., 2021; von Helversen et al., 2018). While these tasks are well suited to observing exploratory behavior in decision-making, exploration as seen from an evolutionary perspective takes place in a spatial context instead of an abstract space. This type of exploration is measured in spatial exploration paradigms and so far has been mostly investigated in animal studies. Here, the animal is usually exposed to an unknown environment, and exploration behavior is quantified by analyzing its movement trajectory during exploration (Belzung, 1999; Freund et al., 2013; Kalueff et al., 2007; Paulus & Geyer, 1993; Paulus et al., 1990; Rosenberg et al., 2021).

Studies on human movement behavior often use real-life observational data of everyday mobility (Alessandretti, Sapiezynski, et al., 2018; Bongiorno et al., 2021; Müller et al., 2022). However, such data might not be ideal for the investigation of exploration behavior, as people often move through familiar spaces (for example, the daily commute to workplaces or universities) rather than unknown environments. Experimental paradigms that focus specifically on exploration behavior therefore mostly use clearly defined real-life environments like single rooms, or mazes or virtual environments. In this type of study, there is large heterogeneity in terms of the kind of exploration behavior that is investigated. In the human literature, exploration is often linked to concepts like *foraging*, *searching, wayfinding*, or *navigation* (Reader, 2015; Wiener et al., 2009). These constructs relate to extrinsically motivated goal-directed exploratory behavior, which is often characterized through performance metrics like time-to-goal or by comparing observed trajectories to optimal routes. In contrast, methods for the characterization of *free exploration* are much scarcer. Free exploration has been defined as undirected, intrinsically motivated exploration behavior (Berlyne, 1960; Gottlieb et al., 2013; Hughes, 1997; Wiener et al., 2009). Since (in contrast to goal-directed exploration) it lacks obvious performance metrics, it is not trivial to quantify. While there is a large body of literature on measures of free exploration in animals, these measures have not yet been well defined for human research. In consequence, there is large heterogeneity in the selection of analysis techniques and outcome variables between studies, which makes both the comparison of different human studies and the translation of observations between the human and the animal field very difficult. To tackle this problem, we aim to provide a comprehensive summary of measures that can be used to characterize both goal-directed and free spatial exploration in humans.

**Fig. 1:**
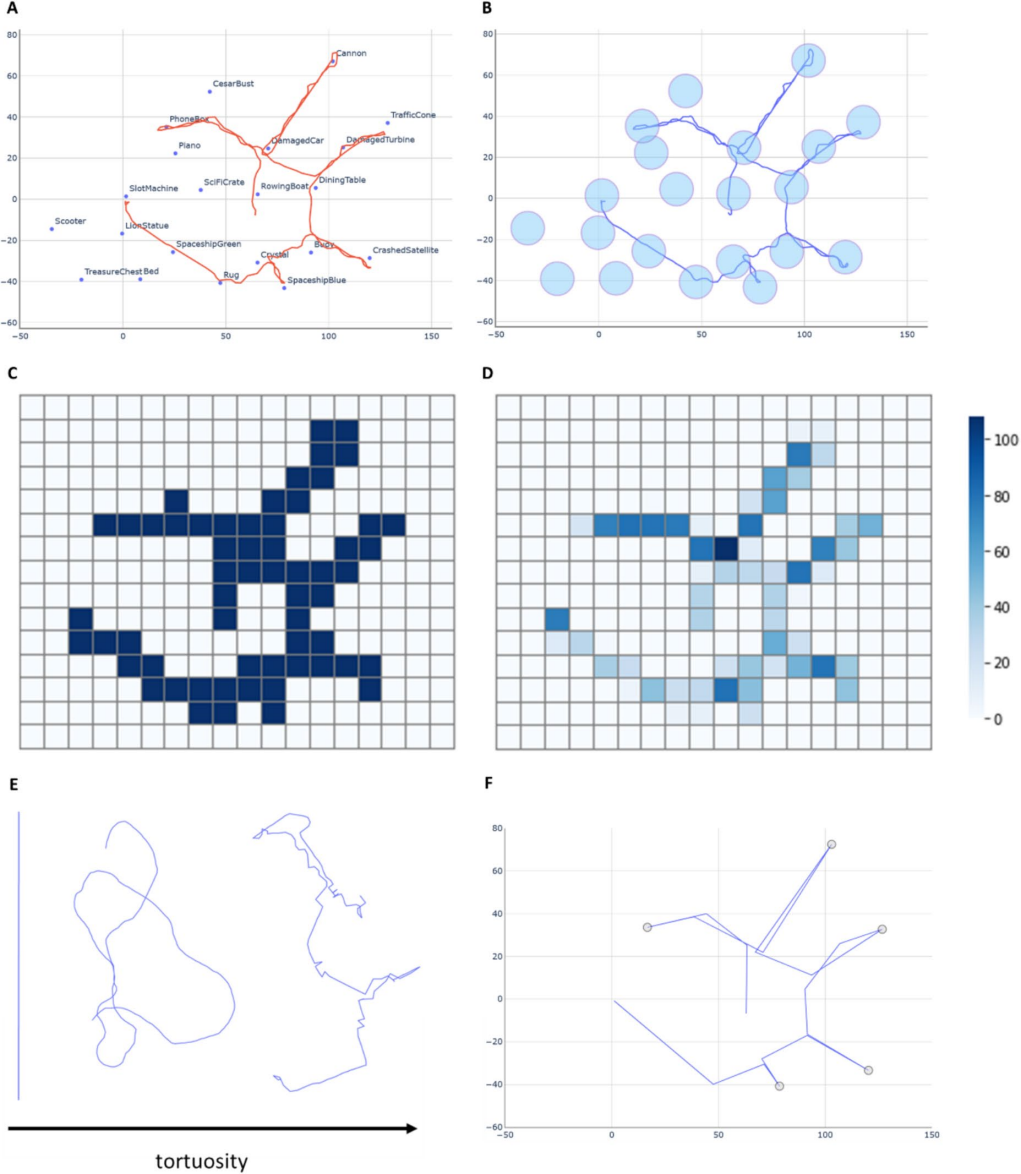
Visualization of the different exploration measures proposed in the current article (all trajectories from the NEMO dataset). For each participant, the raw data give a two-dimensional trajectory (**A**). *Landmark Visits* and *Landmark Revisits* can be calculated by defining a regular area around the landmark coordinates (e.g., circles with 10-m radius, shown in blue), while area exploration can be represented as a heatmap showing the frequency of visits to each bin, which is used in the *Area Covered* (**C**) as well as the *Roaming Entropy* calculation (**D**). Tortuosity as measured by *Fractal Dimension* or *Sinuosity* increases the more a trajectory deviates from a straight line (**E**). The trajectory resampled to the flight scale reveals *Turnarounds* (**F**, turnarounds marked by gray circles), which have been suggested to represent less efficient exploration behavior

Importantly, exploration trajectories represent a type of time series data. However, the spatial nature of movement trajectories implies that typical techniques for the analysis of time series data (Fulcher et al., 2013; Lubba et al., 2019) often fall short, since they usually focus on one dimension across time (e.g., voltage in an electrocardiogram). Therefore, we first focused on measures that have already been used in the context of trajectory analysis and have been associated with a specific behavioral meaning. Crucially, this implies that we did not look at approaches that classify trajectories through machine learning methods (for example, as shown in Bian et al., 2018, and Dubois et al., 2021). Second, we predominantly selected measures that have been used to analyze movement in the context of relatively enclosed spaces rather than completely open terrain (thus ignoring measures like the mean squared displacement or the straightness index, which carry less meaning if movement is confined). Furthermore, as many experiments on human exploration use virtual rather than real-life environments, we also decided to not incorporate measures based on movement speed (as its meaning in virtual environments can be very arbitrary). Lastly, to ensure applicability across as many contexts and paradigms as possible (e.g., GPS data as well as data collected in virtual environments), we focused on measures that can be computed from two-dimensional trajectories (Fig. 1A). The collected measures are summarized in Table 1, with additional graphical representations in Fig. 1.

**Table 1:** Summary of exploration measures

Measure	Meaning	References
Path length	Total length of the trajectory	(Clemenson et al., 2019; Farran et al., 2022; Meade et al., 2019)
Pausing	Time spent without movement	(Gagnon et al., 2016)
Area covered	Area covered during exploration	(Baumann et al., 2020; Farran et al., 2022), similar to *Daily Path Area* in (Šimon et al., 2019)
Roaming entropy	Distribution of the frequency of movement across the area	(Brunec et al., 2022; Cen et al., 2022; Clemenson et al., 2019; Särkelä et al., 2009; Schomaker et al., 2022)
Minimum convex polygon	Area of the smallest polygon that contains all data points	(Šimon et al., 2019)
Fractal dimension	Tortuosity of the trajectory	(Henry et al., 2010; Kearns et al., 2010, 2011; Perry et al., 2009; Yaremych et al., 2019; Young et al., 2007)
Sinuosity*	Tortuosity of the trajectory	(Brudzynski & Krol, 1997)
Landmark visits	Number of landmarks visited	(De Alencar et al., 2015; Fornasari et al., 2013)
Landmark revisits	Number of returns to already visited landmarks	(Fornasari et al., 2013)
Revisiting	Average number of returns to already visited places	(Gagnon et al., 2016, 2018; Munion et al., 2019)
Turnarounds	Number of turns with an angle > 180 degrees	(Farran et al., 2022)
Flight turnarounds	Number of turns with an angle > 180 degrees (flight scale)	(Rhee et al., 2011)
Area efficiency*	Efficiency in covering an area	Similar to *Efficiency* in (Rosenberg et al., 2021)
Landmark efficiency*	Efficiency in visiting landmarks	Similar to *Efficiency* in (Rosenberg et al., 2021)

* Measure previously only used in animal studies

One approach towards the characterization of exploration behavior is to measure the extent of exploration, either by how much an individual moves or how its movement is dispersed across an area (Fig. 1C and D). Another possibility is to quantify the tortuosity, or “crookedness,” of the trajectory (Miller et al., 2011, Fig. 1E). Studies also often employ measures to assess the efficiency of exploration. While efficiency is a concept mostly found in goal-directed wayfinding tasks, we can also translate this concept to free exploration. Doing this, we assume that, even for free exploration, there is a latent goal for exploration, which is to collect as much information about the environment as possible (Johnson et al., 2012). The most efficient way to reach this goal is to cover as much area as possible with as few recursions to already known areas as possible (Fig. 1F). Both virtual and real-life environments also usually include landmarks, such as buildings or objects. While exploration towards such points of interest is currently predominantly measured in studies of goal-directed exploration, we propose that intrinsic free exploration is shaped by the landmarks present in the environment as well. Therefore, we also include measures of landmark-oriented exploration here (Fig. 1B). More detail on each of the measures is presented in the supplementary information (SI, section A). Additionally, we provide Python code for their computation, since currently available public software packages for analysis of movement data like *traja* (Shenk et al., 2021) or *trajr* (McLean & Skowron Volponi, 2018) are more focused on the analysis of movement data in general and lack some measures specifically designed to quantify exploration behavior (e.g., measures on area exploration or efficiency).

Another problem caused by the lack of a systematic approach towards the quantification of exploration behavior is that it is currently unknown how the various measures of exploration relate to each other and what facets of exploratory behavior they actually capture. This information is critical to enable informed decisions on how to interpret different measures of exploration behavior. In the animal literature, various studies have investigated this issue (Jähkel et al., 2000; Markel’ et al., 1988; Paulus & Geyer, 1993; Paulus et al., 1999; Tanaka et al., 2012). While there is considerable heterogeneity between studies regarding paradigms, analysis techniques, and measures used, we identified three main components of exploration that consistently emerged across these experiments. The first component, which we here call *locomotor activity*, includes measures that represent pure movement, like the total distance traveled (e.g., *Path Length*). The second component, which we here call *exploratory activity,* refers to measures of the spatial extent and the spatial variability of movement (i.e., *Roaming Entropy*). Third, a component which we here call *spatial shape*, includes measures like *Fractal Dimension* representing the spatial organization of the trajectory. To tackle this second problem, the current study aims to investigate whether these main components of exploration behavior can also be unveiled in human data.

We therefore investigated whether measures of human exploration behavior can be similarly clustered by analyzing a recently collected dataset of 409 human participants who explored one of two different virtual environments (Ruitenberg et al., 2022; Schomaker et al., 2022). For all individual movement trajectories in the dataset, the 14 movement measures summarized above (Table 1), were calculated. To assess whether these measures capture different aspects of exploration behavior, we used a hierarchical cluster analysis. Similar to factors or principal components in other dimensionality reduction methods, each cluster consists of variables that are strongly related to each other and thus represent similar information (Chavent et al., 2012). However, in contrast to factor analysis or principal component analysis, hierarchical clustering allows us to reliably investigate relations between variables even if there is substantial multicollinearity and interdependence between variables or a low number of variables per factor. In accordance with the related research in animals (Jähkel et al., 2000; Kalueff et al., 2007; Markel’ et al., 1988; Paulus & Geyer, 1993; Paulus et al., 1999; Tanaka et al., 2012), we expected three main clusters to emerge: a “Locomotor Activity” cluster (containing *Path Length* and *Pausing*), an “Exploratory Activity” cluster (containing *Area Covered* and *Roaming Entropy*), and a “Spatial Shape” cluster (containing *Sinuosity* and *Fractal Dimension*). Additionally, we explored whether our measures of general exploration efficiency (*Revisiting*, *Area Efficiency, Turnarounds*, and *Flight Turnarounds*) as well as our measures of landmark-oriented exploration (*Landmarks Visited*) and landmark exploration efficiency (*Landmark Revisiting*, *Landmark Efficiency*) would form independent clusters.

For data-driven approaches like hierarchical clustering, it is important to validate that the clusters represent meaningful aspects of exploration behavior that also generalize to other datasets. Here, we therefore applied the same analysis to a second, independent dataset of 102 participants (Brunec et al., 2022) representing goal-directed exploration of a novel environment.

## Methods

### Sample and procedure

**Fig. 2:**
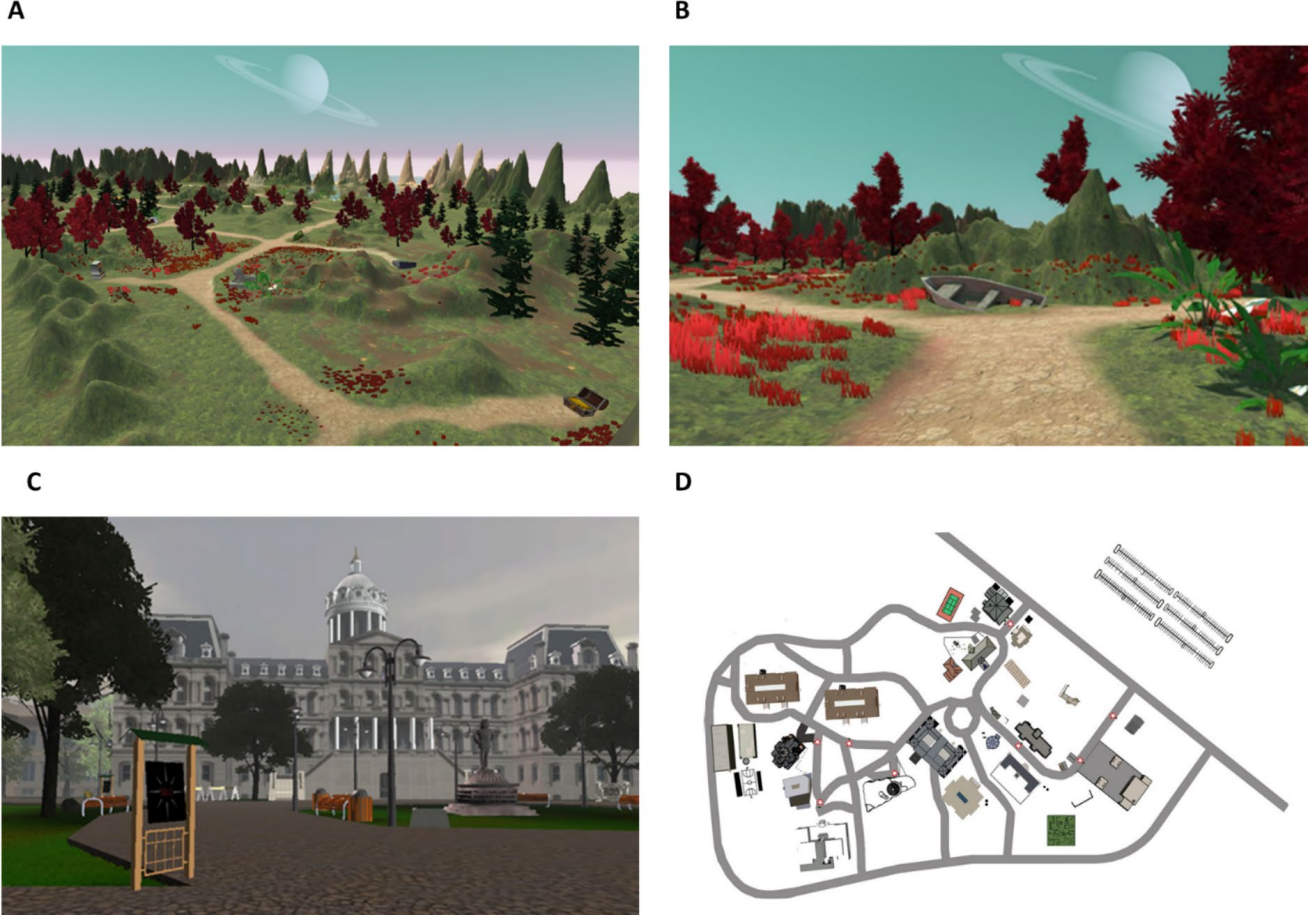
Overview of one of the two virtual environments used in the NEMO dataset (**A**). Twenty landmark objects were positioned throughout the environment at intersections and road endpoints, which participants could explore freely (**B**). In the SILCTON datasets, the map represented a university campus with several buildings (**C**). Here, participants were given the explicit goal of finding eight specifically named buildings (**D**, building position marked by the red stars)

Our first dataset (here called the NEMO dataset) comprised data collected from 487 participants during a large-scale public science experiment in the NEMO Science Center in Amsterdam (Ruitenberg et al., 2022; Schomaker et al., 2022). Participants’ age ranged from 8 to 77 years (see the SI, section B, as well as Schomaker et al., 2022) for a more detailed description of the sample). The dataset consists of exploration data in two virtual environments that were created in Unity (version 2017.2.21f1) and matched in size, path length, and number of intersections. Both environments represent fantasy islands with different objects as landmarks (such as a treasure chest), including land and a body of water (Fig. 2A and B). Participants could move in all four directions using the WASD keys on the keyboard, jump using the spacebar, and determine the heading direction via their mouse. The speed of movement was fixed and could not be altered by the participants. Participants explored one of the two virtual environments for 150 s (environments were allocated at random to each participant) and were instructed that they could navigate freely but should try to stay on the paths (note that it was nevertheless possible to also explore the area off the paths). Since no other tasks were given, exploration could be considered as “intrinsic exploration” as defined by Berlyne (1960). Environments were presented on one of six laptops running on Windows 10 (Microsoft, 2015). During exploration, the *X*, *Y*, and *Z* coordinates of the player position were logged with a sampling rate of approximately 15 Hz (but deviations from this sampling rate occurred, see “Data Preprocessing” in the supplementary information).

In the original study, participants also performed a series of other tasks (including a second round of exploration, a word learning task, a motor learning task, and a landmark memory test). However, this article only focuses on the first exploration round. The experimenter stayed in the testing room throughout the entire procedure to start the tasks and to answer questions. For more detailed information on the experimental procedure see the SI, section C.

The second dataset (here called the SILCTON dataset) consisted of data from two experiments by Brunec et al. (2022) including 136 university students as participants (no detailed age information available). Here, the environment consisted of a virtual university campus created with the Unity engine (the Virtual Silcton environment, Weisberg & Newcombe, 2020). Similar to the NEMO dataset, participants could move in all four directions using the keyboard and could adjust their heading direction by moving the mouse. Compared to the NEMO environment, the SILCTON environment was roughly 20 times larger, and individuals were able to explore much longer (16 min for experiment 1, 25 min for experiment 2). Similar to the NEMO environment, movement speed was set to a fixed value and was of similar magnitude (median step length in the NEMO environment: 0.48, median step length in the SILCTON environment: 0.50). While the exploration experience was continuous in experiment 2, experiment 1 consisted of four blocks of 4 min of exploration, alternating with 1 min of a specific task (map sketching, landmark recognition, coloring book, continuous exploration—see Brunec et al., 2022) for which movement was paused and then continued at the same position. Crucially, participants in both SILCTON experiments were instructed to search for eight specific buildings on the virtual campus (Fig. 2C and D). Therefore, the SILCTON dataset, in contrast to the NEMO dataset, represents goal-directed exploration.

### Data preprocessing

#### Preprocessing for the NEMO dataset

Out of 487 participants in the original NEMO dataset, 55 were excluded due to either technical problems or other issues regarding correct performance of the task (e.g., not speaking Dutch or English as their first language, being on the phone during the experiment, or getting help from a nearby person). This resulted in 432 participants with a valid dataset for the first exploration session. From these, we excluded seven participants because they fell off the boundaries of the map during exploration (*y* coordinate ≤ 100), and another 16 were excluded as they showed disproportionally low movement activity (only moved in less than one fifth of the available time, resulting in less than 30 s of movement in total), which may reflect failure to understand the controls or distraction. The final sample consisted of 409 participants (*median age* = 23.83, *SD* = 16.47, *male:female:other* = 211:195:3).

Since the original experiment was conducted on different laptop setups that did not provide timestamps, we controlled whether coordinates were registered regularly. The number of logged data points was used to retroactively calculate the original individual sampling rates by dividing the number of data points logged (e.g., 3000) by the total play time (150 s, constant across all participants). The resulting sampling rates varied among participants, ranging from ~ 50 to ~ 10 Hz, which was likely caused by the environments running on laptops with different computational capabilities. However, all datasets still had sufficiently high sampling rates to detect any meaningful changes in player movement (all sampling rates ≥ 10 Hz). Next, we checked for within-session variability in sampling rate. As the movement speed was fixed throughout the task, we used variability in step length as a proxy of variability in sampling rate. To assess variability in step length, we computed the coefficient of variation of step lengths for each trajectory (using only steps with a length > 0). This allowed us to compare the relative variability of step lengths across trajectories independently of the absolute step length values. No dataset showed a disproportionally high variability in step lengths (cutoff: coefficient of variation > 3 * interquartile range). In addition, we controlled for disproportionally high step lengths as an indicator of large lags. A “large lag” was defined as a step length greater than three times the median step length in the respective trajectory (considering only steps with a length > 0). The exclusion criterion was set at a percentage of lag steps > 0.1%, but the highest percentage of lag steps found was 0.03%.

Since some measures (i.e., *Path Length*, *Revisiting*, *Roaming Entropy*) are sensitive to differences in sampling rates, we resampled all trajectories to a common sampling rate of 10 Hz (the slowest observed individual sampling rate) using the *trajr* temporal resampling algorithm (McLean & Skowron Volponi, 2018). As a final data preprocessing step, we trimmed the beginning of all trajectories up to the first movement to remove the initial idle time where participants still listened to the instructions given by the experimenter (*median* = 16.7 s, *SD* = 10.73 s).

#### Preprocessing for the SILCTON dataset

From 130 participants in the original SILCTON dataset (78 participants from experiment 1 and 52 from experiment 2), the authors provided us with the data for 51 participants from experiment 1 and 52 participants from experiment 2, thus creating a set of 103 trajectories. From these, one subject was excluded due to a much shorter logging time (13.7 min versus a median exploration time of 26.7 min). Regarding sampling rate variability, we applied the same procedure as for the NEMO dataset. One participant showed a disproportionally high variability in step length. However, since this value still was very low (about one fifth of the median step length), we excluded no further participants and therefore obtained a final dataset of 102 trajectories (*male:female* = 42:60). Aside from gender, the dataset contained no information on age or any other individual characteristics. However, most participants were university students and all were above the age of 18.

### Extraction of exploration measures

We extracted all exploration measures from the preprocessed trajectories. For detailed descriptions and the single measures, please refer to the SI, section A. Since some measures require individualized parameter settings (e.g., *bin size* in the *Roaming Entropy* calculation), the problem of finding sensible parameter values arises. Here, we followed a two-step process to find adequate parameter values. First, we selected an initial starting value, preferably based on either previous literature or specific characteristics of the environment (e.g., the distance traveled in a certain amount of time, or distances between landmarks). Second, we evaluated this initial parameter by iterating across a range of both smaller and larger parameter values and analyzing the resulting parameter distributions. From these data, we then selected the most optimal value. For more details on the parameter selection procedure and the data which informed our final parameter settings, see the SI, section D.

For *Area Covered* and *Roaming Entropy*, we used a bin size representing an area of 14 × 14 (15 × 15 for the SILCTON environment) virtual meters (vm). The normalization parameter *k* in *Roaming Entropy* was set to the total size of the environments (maximal amount of unique bins explored by all subjects). For *Fractal Dimension*, we chose 20 step lengths, starting at half the median step length and increasing to 10 times the median step length. For the calculation of *Sinuosity*, we chose the original formula by Bovet and Benhamou (1988) for trajectories with regular step lengths. We therefore computed a re-discretized version of each trajectory using the *rediscretize()* function of the *traja* package (Shenk et al., 2021). The step length for re-discretization was set to *r* = 0.48 (0.50 for the SICLTON environment), which corresponded to the respective median step length across all trajectories. For *Landmark Visits* and *Landmark Revisits*, a landmark was considered visited if a participant moved within a circular area with radius *r* = 20 vm centered around that landmark. For *Revisiting*, we used a radius of* r* = 14 vm.

We calculated *Turnarounds* using the 180-degree angle cutoff suggested by Farran et al. (2022), with angles ranging from 0 (no change in heading direction) to 180 degrees (complete reversal of heading direction). Previously, this measure was proposed as a measure of efficiency (Farran et al., 2022), as all occasions in which participants reverse their heading direction for 180 degrees and thus retrace their own path are counted. However, it can be difficult to calculate turnarounds from the raw movement data, as small movements (i.e., strafing in virtual environments) can produce a high number of turnarounds, while the overall heading direction remains stable. We propose that a more reliable representation of turnarounds can be achieved by looking at the trajectory not on the scale of steps, but on the scale of flights (Fig. 1F). While a step simply represents the distance between two consecutive data points, a flight summarizes all steps with a continuous movement direction, given a predetermined degree of deviation (Rhee et al., 2011). Resampling a trajectory to the scale of flights removes small-scale movements of the trajectory’s signal. In contrast to the original *Turnarounds* measure, this *Flight Turnarounds* measure therefore should reflect more long-term changes in heading direction. For *Flight Turnarounds*, we resampled each trajectory to the scale of flights using the Ramer–Douglas–Peucker (RDP) algorithm (Hirschmann, 2016). The maximum distance for line simplification in RDP was set to *ε* = 6, indicating that the estimated flights were allowed to deviate from the actual data points by up to six virtual meters. All turning angles ≥ 160 degrees were subsequently counted as turnarounds. Compared to prior research (Farran et al., 2022), we allowed for a wider range of angles to be classified as turnarounds by choosing a cutoff of 160 degrees instead of 180 degrees. This procedure was based on the observation that on the flight-based trajectory, very few turnarounds that could be visually identified as turnarounds actually showed values of precisely 180 degrees, but rather varied between 160 and 180 degrees. All preprocessing procedures and feature calculations were performed in Python (version 3.9.7) using Spyder (version 5.1.5). For more details on the single measures, see the SI (section A).

### Statistical analysis

We standardized all variables prior to conducting the hierarchical clustering analysis to remove any effects of variable scale. For each dataset, we then ran a hierarchical clustering algorithm using the R package *ClustOfVar* (Chavent et al., 2012). As a similarity measure, *ClustOfVar* uses squared Pearson correlations, so the similarity of variables is quantified independently of the direction of their correlations. The number of clusters was determined by visual inspection of the dendrogram as well as the plot of the height of the aggregation levels. For the latter, we additionally applied the *kneedle* algorithm as an objective elbow detection method (Satopaa et al., 2011).

Since the age distribution in the NEMO dataset was quite spread out (8 to 75 years—see the SI, section B), we additionally investigated whether the clustering result was dependent on participant age. Therefore, we ran the clustering procedure two more times for both a younger and an older age group. We chose age ranges in a way that allowed us to keep a roughly equal number of participants in each subgroup (“young group”: 8–14 years, *n* = 213; “old group”: 15–75 years, *n* = 207). We found no relevant age group-related changes to the cluster structure (see the SI, section E).

All statistical analyses were run in R (version 3.6.3) using RStudio (version 2022.7.1.554). Original data and the code to recreate preprocessing, feature extraction, and the statistical analyses are available on https://github.com/valentinbaumann/explorationMeasures.

## Results

### Cluster analysis (NEMO)

**Fig. 3:**
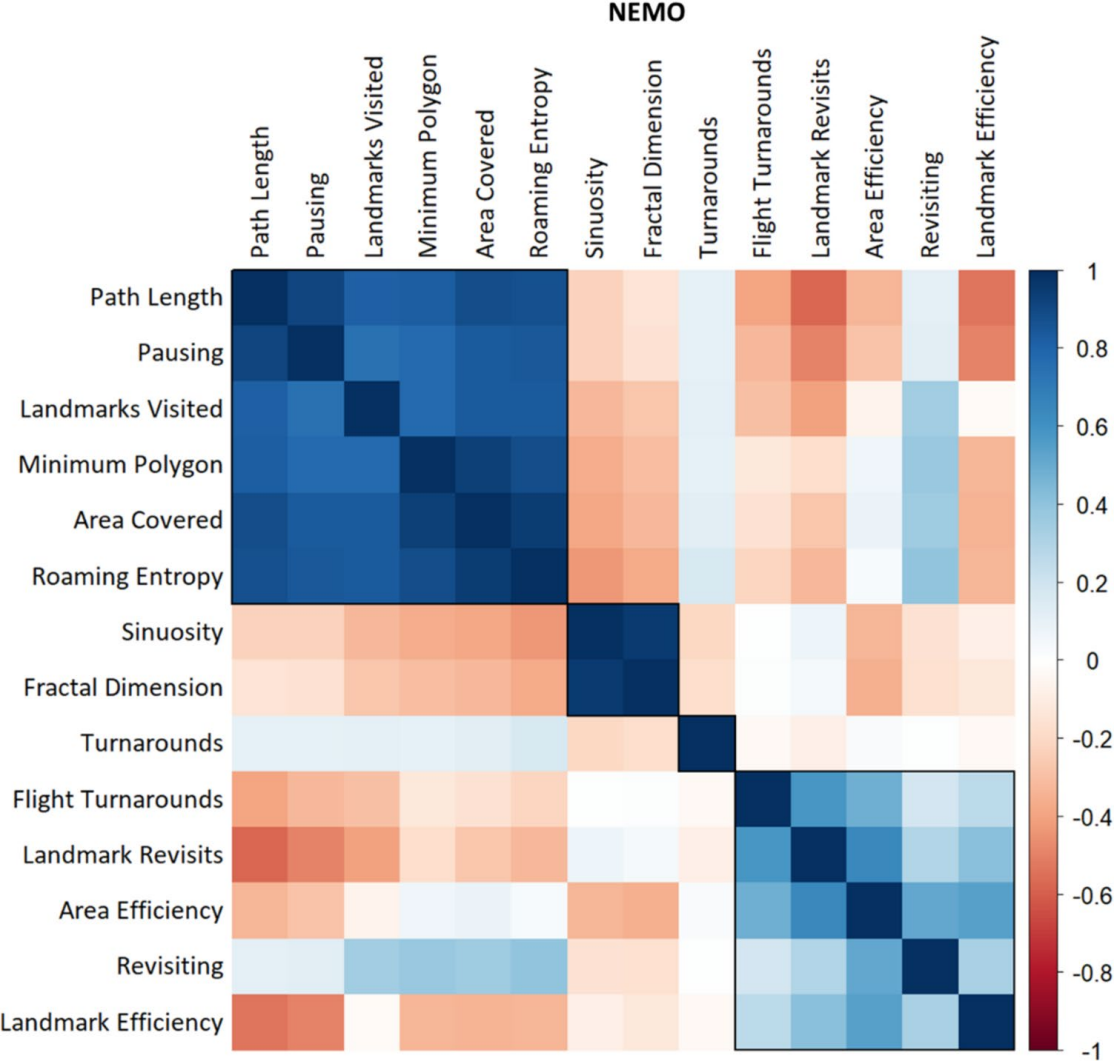
Similarity matrix for all investigated measures of exploration behavior in the NEMO dataset (note that while *ClustofVar* uses the squared Pearson correlation as similarity measure, the plot shows the standard Pearson correlation for better interpretability). The rectangular boxes represent the three main clusters determined by our hierarchical cluster analysis, plus a potential fourth cluster consisting of the original *Turnarounds* measure. We inverted the measures *Pausing*, *Revisiting*, and *Landmark Revisits* as well as *Turnarounds* and *Flight Turnarounds* for this plot to ensure consistent meaning in respect to the other measures (higher score = higher exploratory behavior/higher efficiency)

**Fig. 4:**
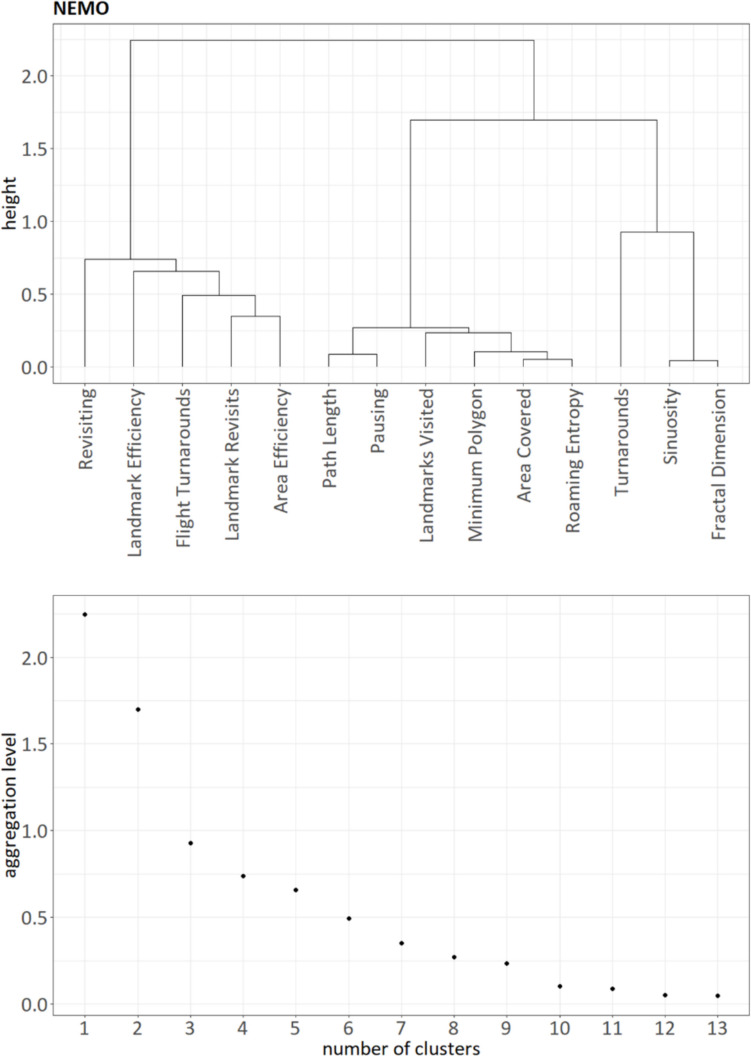
Results of the hierarchical clustering shown as a dendrogram (**A**) and as a plot of the aggregation levels (**B**) for then NEMO dataset. Measures that join at a lower height in a dendrogram are more strongly related to each other than measures that join at greater height. On visual inspection of the dendrogram (**A**), three main clusters emerge (left branch: cluster “Exploration Efficiency,” middle branch: cluster “Exploratory Activity,” right branch: cluster “Spatial Shape”). Using the elbow method and the kneedle algorithm, the plot of the height of the aggregation levels versus the number of variables suggests a number of three to four clusters (**B**). However, note that the original *Turnarounds* measure does not seem to be closely related to any of the other measures (**A**, also see Fig. 3). While this measure could be interpreted as a singular fourth cluster, we decided to not include it in our further analysis as it does not represent its intended meaning (Fig. 5 and main text)

For the NEMO dataset, visual inspection of the similarity matrix (Fig. 3), the dendrogram (Fig. 4A), and the height of aggregation levels (Fig. 4B) indicated that our clustering procedure resulted in three main clusters, with the possibility of a very small fourth cluster. The *kneedle* algorithm suggested a number of four clusters. Cluster 1 contained the variables *Path Length*, *Pausing*, *Area Covered*, *Roaming Entropy*, and *Minimum Polygon*. Similarly to the SILCTON dataset, we labeled this the “Exploratory Activity” cluster. Cluster 2 comprised *Sinuosity* and *Fractal Dimension* and was again labeled the “Spatial Shape” cluster. Cluster 3 consisted of *Revisiting*, *Landmark Revisits*, *Flight Turnarounds*, *Area Efficiency*, and *Landmark Efficiency*. We labeled this cluster “Exploration Efficiency.”

**Fig. 5:**
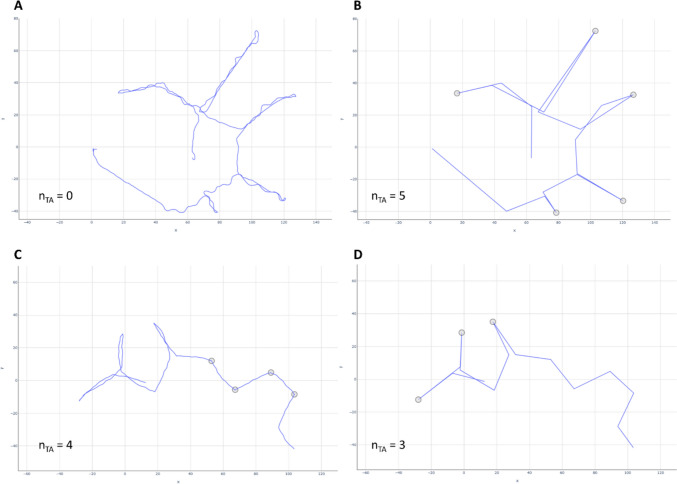
Comparison of *Turnarounds* using the original method by Farran et al. (2022) and our proposed improved method (*Flight Turnarounds*). The goal of both methods is to quantify how often participants turn around and retrace their previous path by analyzing the turning angles. Turning angles classified as turnarounds are shown as gray dots. Our data show that the original *Turnarounds* method cannot reliably detect turnarounds that are relatively easy to discern visually (**A**), while *Flight Turnarounds* correctly identifies all five turnarounds (**B**). At the same time, *Turnarounds* can be generated by comparatively small-scale movement that is not indicative of actual path retracing (**C**), which is not an issue when angles are computed on the flight scale. n_TA_ = number of turnarounds

The fourth cluster included *Turnarounds* as a single measure. Interestingly, contrary to its intended meaning as an index of efficiency, it did not show any significant correlation to the measures in Cluster 3 (Fig. 3). As the original computation of *Turnarounds* might be problematic to its focus on small-scale movement (see Sect. "Extraction of exploration measures" *Extraction of exploration parameters*), we further investigated whether both *Turnarounds* and *Flight Turnarounds* correctly measured the intended behavior of path retracing. We observed that the original *Turnarounds* computation often was not able to capture all turning points, while the proposed alterative of *Flight Turnarounds* showed much better performance (Fig. 5). Consequently, we decided to dismiss *Turnarounds* from further analysis and accepted the three main clusters of “Exploratory Activity,” “Spatial Shape,” and “Exploration Efficiency” as the clustering result. The loadings of each measure on the three final clusters are shown in Table 2. Note that we repeated the entire clustering procedure without the original *Turnarounds* measure to obtain clean cluster loadings without the influence of *Turnarounds*.

**Table 2:** Cluster loadings for each exploration behavior measure (NEMO)

Measure	“Exploratory Activity”	“Shape”	“Exploration Efficiency”
Path length	**0.95**	− 0.20	− 0.49
Pausing	**0.91**	− 0.20	− 0.42
Area covered	**0.97**	− 0.36	− 0.10
Roaming entropy	**0.96**	− 0.41	− 0.14
Minimum polygon	**0.93**	− 0.34	− 0.10
Landmarks visited	**0.89**	− 0.30	− 0.14
Fractal dimension	− 0.35	**0.99**	− 0.17
Sinuosity	− 0.28	**0.99**	− 0.14
Landmark revisits	− 0.40	0.01	**0.82**
Revisiting	0.31	− 0.16	**0.60**
Flight turnarounds	− 0.27	0.01	**0.88**
Area efficiency	− 0.09	− 0.35	**0.68**
Landmark efficiency	− 0.37	− 0.11	**0.69**

Pearson correlations of exploration measures and the three synthetic cluster variables generated by the hierarchical clustering procedure. Bold-faced numbers indicate the measures belonging to the respective cluster. Note that we inverted the measures *Pausing, Revisiting, Landmark Revisits, *and* Flight Turnarounds* for this table to ensure consistent meaning in respect to the other efficiency measures (higher score = higher efficiency), while the original *Turnarounds* measure was excluded

### Cluster analysis (SILCTON)

**Fig. 6:**
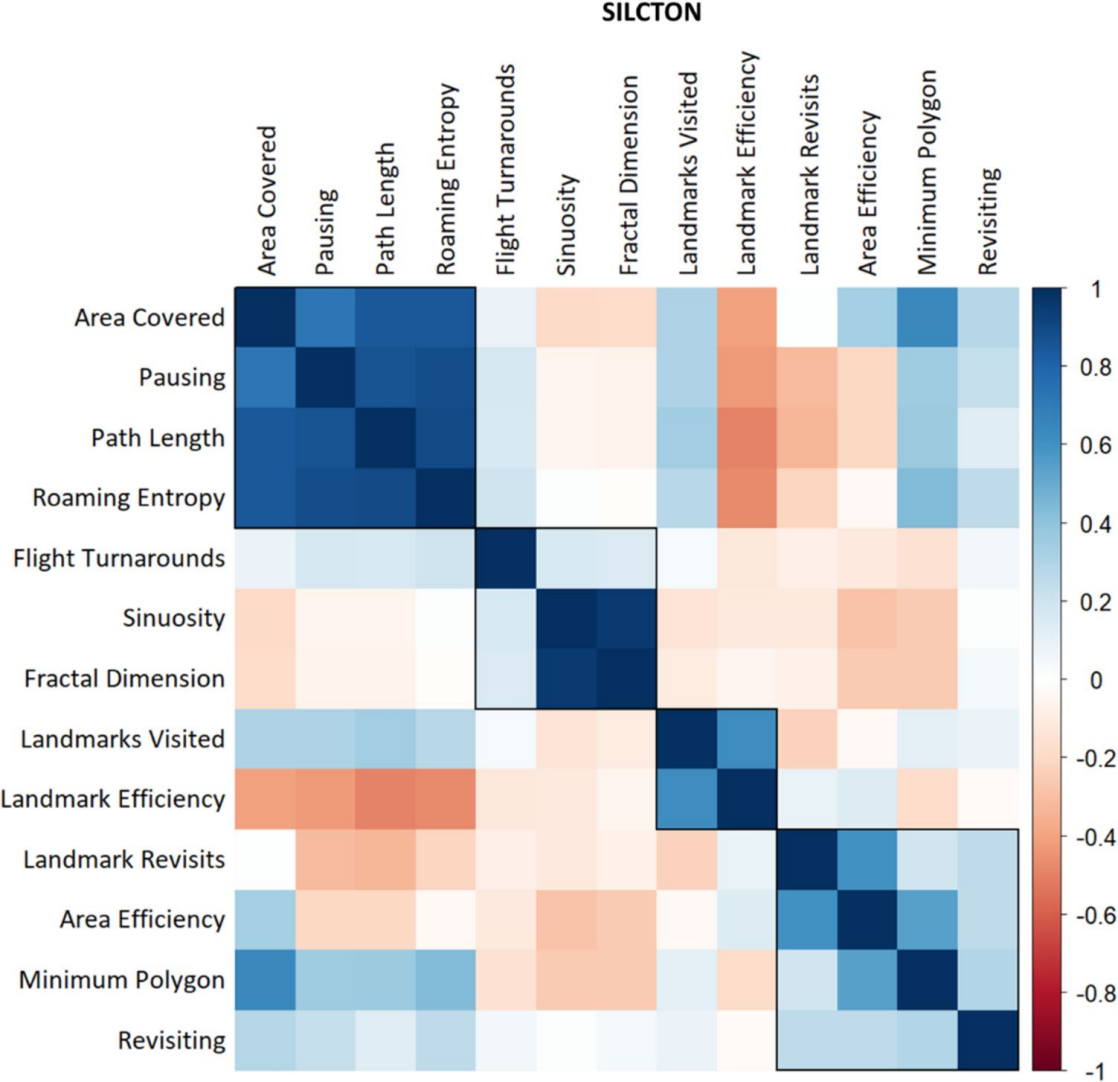
Similarity matrix for all investigated measures of exploration behavior in the SILCTON dataset, showing the four clusters. Note that while *ClustofVar* uses the squared Pearson correlation as similarity measure, the plot shows the standard Pearson correlation for better interpretability. We inverted the measures *Pausing*, *Revisiting*, and *Landmark Revisits* for this plot to ensure consistent meaning in respect to the other measures (higher score = higher exploratory behavior/higher efficiency). Note that this time we did not invert *Flight Turnarounds*, as the clustering suggested it represented a measure of *Spatial Shape* rather than *Exploration Efficiency* (more turnarounds = more tortuous pathing)

**Fig. 7:**
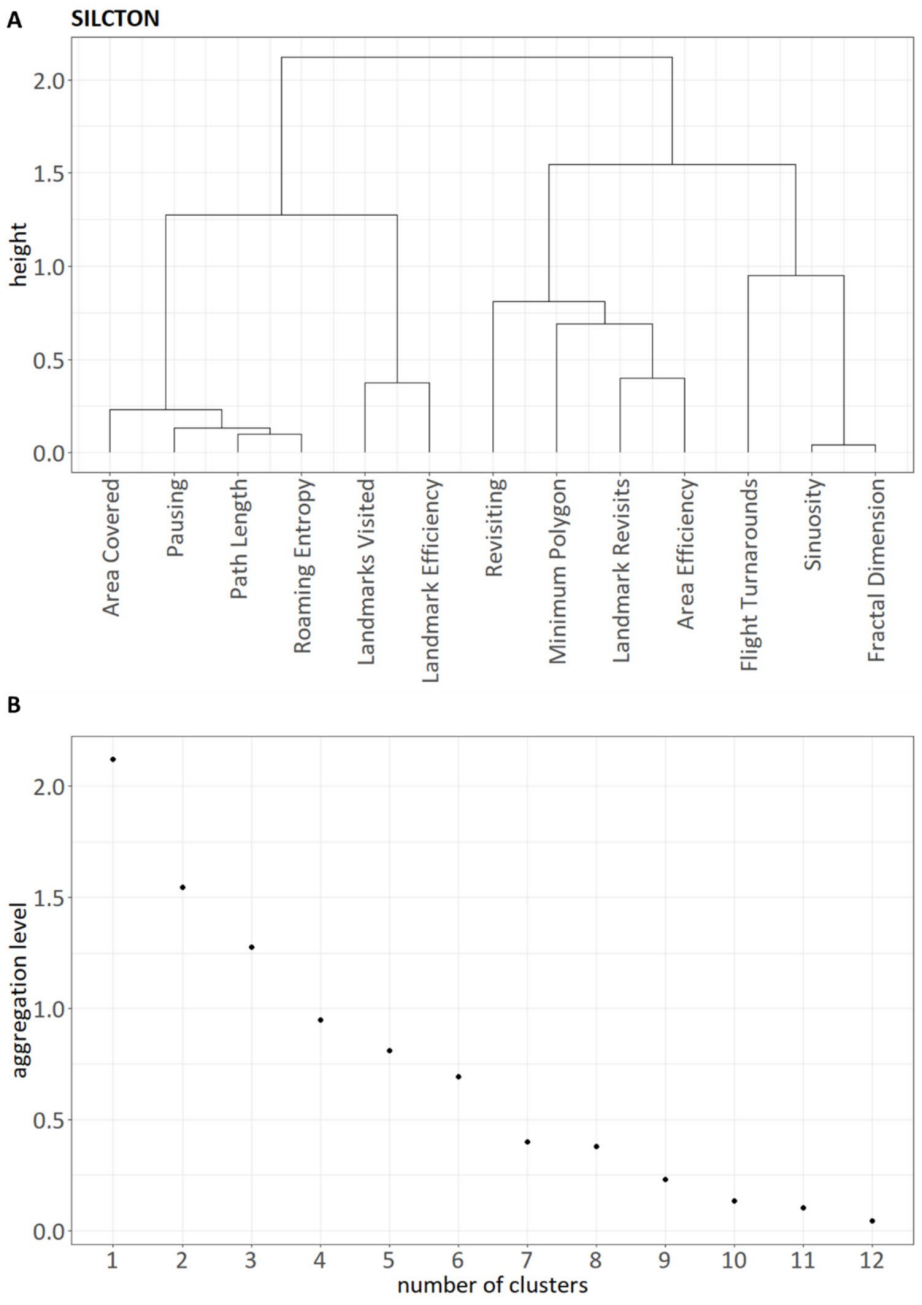
Results of the hierarchical clustering shown as a dendrogram (**A**) and as a plot of the aggregation levels (**B**) for the SILCTON dataset. Four main clusters emerge (outer left branch: cluster “Exploratory Activity,” middle left branch: cluster “Goal Efficiency,” middle right branch: “Area Efficiency,” outer right branch: cluster “Spatial Shape”). Note that while the *Flight Turnarounds* measure was grouped with Sinuosity and Fractal Dimension, it did not seem to be closely related to these variables or to any of the other measures (**A,** also see Fig. 6). Using the elbow method and the kneedle algorithm, the plot of the height of the aggregation levels versus the number of variables also suggests a number of four clusters (**B**)

Next, we ran the same procedure for the SILCTON dataset. Note that, similar to the previous analysis, we left out the *Turnarounds* measure. Again, we inspected the similarity matrix (Fig. 6), the dendrogram (Fig. 7A), and the height of aggregation levels (Fig. 7B). The dendrogram suggested four main clusters, with the possibility of a very small fifth cluster. While on visual inspection the plot of the aggregation levels showed no clearly visible elbow, the *kneedle* algorithm suggested a number of four clusters. We therefore selected four clusters as the final solution.

Cluster 1 contained the variables *Path Length*, *Pausing*, *Area Covered*, and *Roaming Entropy*. Similarly to the NEMO dataset, we labeled this the “Exploratory Activity” cluster. Cluster 2 comprised *Sinuosity* and *Fractal Dimension* and again was labeled the “Spatial Shape” cluster. Cluster 3 consisted of *Revisiting*, *Landmark Revisits*, *Area Efficiency*, and *Minimum Polygon*. We labeled this cluster “Area Efficiency.” Cluster 4 contained both *Landmarks Visited* and *Landmark Efficiency*. As these variables mostly targeted goal-directed exploration, we labeled this cluster “Goal Efficiency.” In contrast to the NEMO dataset, *Flight Turnarounds* did not cluster with other efficiency measures, but was related to the “Spatial Shape” cluster. However, the low correlations of *Flight Turnarounds* with *Sinuosity* (*r* = 0.16) and *Fractal Dimension* (*r* = 0.15) suggested that this relationship was rather loose.

## Discussion

In this study, we aimed to establish a systematic approach towards the characterization of free exploration behavior in humans. We first identified the most common movement measures currently used in human research on spatial exploration from the literature and introduced refinements of existing measures to capture exploration efficiency more accurately.

Next, we analyzed the different measures of exploration behavior using the NEMO dataset (*n* = 409) to evaluate components of human exploration behavior. Hierarchical clustering revealed three main clusters of exploration measures (Fig. 4). The first cluster included the measures *Path Length*, *Pausing*, *Area Covered*, *Roaming Entropy*, *Minimum Polygon*, and *Landmarks Visited*. Since all of these measures reflected the extent of movement activity related to exploration of the area, we labeled this cluster “Exploratory Activity.” High intercorrelations of the single measures (Fig. 3) as well as high correlations with the synthetic cluster variable (Table 2) indicate that this cluster is highly homogeneous, which suggests that all measures reliably represent the same aspect of exploration. Interestingly, the extent of exploration towards points of interests (*Landmarks Visited*) was strongly related to exploratory behavior in general (Table 2) and did not form a cluster on its own. One reason for this might be that in our environments, the landmarks were very evenly distributed across the area, so a higher level of landmark exploration also necessarily implied greater area exploration. Another possible explanation is that since the NEMO dataset represents free exploration, participants were not specifically focused on moving towards preselected points of interest, but rather explored the area as a whole.

The second cluster represented the spatial shape of the trajectory (straight line vs. tortuous shape, as measured by *Fractal Dimension* and *Sinuosity*) and was therefore labeled the “Spatial Shape” cluster. Similar to the first cluster, the two measures load very high on the synthetic variable (Table 2) and show high intercorrelations (Fig. 3), again suggesting a reliable and homogeneous representation of this facet of exploration behavior.

Our third observed cluster incorporated all measures associated with the efficiency of movement through an area or towards certain points of interests (*Area Efficiency*, *Revisiting*, *Flight Turnarounds, Landmark Revisits*, *Landmark Efficiency*). In contrast to the very homogeneous “Exploratory Activity” and “Shape” clusters, we observed this cluster, termed “Exploration Efficiency,” to be more heterogeneous, as suggested by lower correlations among the cluster’s measures as well as by lower correlations of the cluster’s measures and the synthetic cluster variable (Fig. 4 and Table 2). This indicates that these measures only partially reflect a common aspect of exploration, with a substantial contribution of other sources of variance. Interestingly, most measures also seem to contain their own unique variance components, as suggested by the relatively low correlations among themselves (Fig. 4). One explanation for this might be that, within the group of participants, some individuals could have shown different aspects of efficiency depending on whether their aim was to cover ground (i.e., *Area Efficiency, Revisiting*) or to seek out points of interest (i.e., *Landmark Efficiency, Landmark Revisits*).

As a data-driven method, hierarchical clustering is heavily dependent on the input data. We therefore validated our clustering result using a second set of trajectories (the SILCTON dataset). Here, in contrast to the NEMO data, we observed four instead of three main clusters of exploration. The first cluster included the measures *Path Length*, *Pausing*, *Area Covered*, and *Roaming Entropy*. As this was very close to the first cluster of the NEMO dataset, we again labeled this the “Exploratory Activity” cluster. Similarly, the “Spatial Shape” cluster contained the same two measures as in the previous analysis (*Sinuosity* and *Fractal Dimension*). Again, high intercorrelations of these measures (Fig. 6) as well as high correlations with the respective synthetic cluster variable (Table 3) indicate that these two measures are highly homogeneous and reliably represent the same aspect of exploration.

**Table 3:** Cluster Loadings for each exploration behavior measure (SILCTON)

Measure	“Exploratory Activity”	“Shape”	“Area Efficiency”	“Goal Efficiency”
Path Length	**.96**	–.04	–.04	–.08
Pausing	**.92**	–.03	–.01	–.07
Area Covered	**.91**	–.17	.43	–.06
Roaming Entropy	**.97**	.03	.12	–.12
Fractal Dimension	–.10	**.98**	–.20	–.09
Sinuosity	–.10	**.98**	–.24	–.14
Flight Turnarounds	.17	**.31**	–.11	–.05
Landmark Revisits	–.23	–.10	**.73**	–.07
Revisiting	.27	.04	**.56**	.03
Area Efficiency	–.03	–.28	**.87**	.06
Minimum Polygon	–.47	–.27	**.70**	–.04
Landmarks Visited	.33	–.12	–.03	**.90**
Landmark Efficiency	–.48	–.10	.02	**.90**

Pearson correlations of exploration measures and the four synthetic cluster variables generated by the hierarchical clustering procedure. Bold-faced numbers indicate the measures belonging to the respective cluster. Note that we inverted the measures *Pausing*, *Revisiting* and *Landmark Revisits* for this table to ensure a consistent meaning in respect to the other efficiency measures (higher score = higher efficiency). Note that this time we did not invert *Flight Turnarounds*, as the clustering suggested it represented a measure of *Spatial Shape* rather than *Exploration Efficiency* (more turnarounds = more tortuous pathing)

The third cluster in the SICLTON dataset comprised the *Area Efficiency*, *Revisiting*, *Landmark Revisits*, and *Minimum Polygon* measures. As Area Efficiency was the highest-loading variable on the synthetic cluster score (Table 3), and since most of the measures seemed to be related to the general efficiency of exploration, we termed this cluster the “Area Efficiency” cluster. In contrast, the fourth cluster incorporated both *Landmark Visits* and *Landmark Efficiency*, which measure the exploration of landmarks rather than the exploration of a general area. This is in contrast to the NEMO dataset, where all efficiency measures emerged as a single cluster and might reflect a difference in the type of exploration task. Since the NEMO experiment represents free exploration, it is possible that these participants focused on the exploration of the general area, rather than on specific landmarks. In contrast, the participants in the SILCTON dataset were instructed to search for certain landmarks, and therefore might have adopted a more goal-oriented exploration style.

Despite the task instruction, the two experiments also differed in the time spent exploring as well as their area sizes. However, we consider this a less likely explanation for the differentiation between efficiency types. For one, in addition to the longer exploration time, the SILCTON experiment also offered a much larger environment to explore, creating a similar ratio between the available exploration time and the time needed to achieve a specific goal as in the NEMO environment. Second, we observed the same differentiation of Area Efficiency and Goal Efficiency when we ran separate analyses for the two different SILCTON experiments (see the SI, section F). As these two also differed greatly in the time spent exploring (experiment 1: 16 min, experiment 2: 25 min), this result indicates that the effect seems to be attributable to the difference in exploration task rather than exploration time.

Interestingly, the measures *Flight Turnarounds* and *Minimum Polygon* also clustered differently between the NEMO and SILCTON datasets. This might have been caused by the strong dependence of both measure on the specific layout of the environment. For example, the NEMO environment offered a relatively large number of dead ends and therefore many possibilities where retracing a large proportion of the own path was possible (see Fig. 1). Therefore, *Flight Turnarounds* in this case represented a measure of efficiency (fewer turnarounds—less retracing—higher efficiency). In contrast, the street network in the SILCTON environment showed more loops (see Fig. 2D). Here, participants potentially did not directly turn around that often, but rather used the loops to return to previously visited places. Interestingly, in the SILCTON dataset, *Flight Turnarounds* was, albeit quite loosely, associated with the Spatial Shape cluster. This indicates that, depending on the environment layout, *Flight Turnarounds* can either represent the efficiency of exploration, or act as an indicator of the spatial shape of the trajectory.

Similarly, the change in the *Minimum Polygon* measure from the Exploratory Activity cluster in the NEMO dataset to the Area Efficiency cluster in the SILCTON experiment could also be caused by the measure’s sensitivity to the specific environment layout. Importantly, *Minimum Polygon* is defined by the location of the outermost data points and therefore is heavily influenced by the overall shape of the trajectory. In contrast, measures like *Area Covered* are not dependent on whether the explored area extends very thinly, but across a large space (creating a larger polygon area), or if the same amount of space is explored in a very compact manner (creating a smaller polygon area).

Taken together, analysis of the SILCTON dataset validated the clusters Exploration Efficiency and Spatial Shape as very stable constructs, which appear to persist across different age ranges as well as different environment layouts, exploration times, and exploration tasks. Additionally, these clusters seem to translate well between rodents and humans, as they correspond well to the different facets of exploration previously observed in the animal literature (Jähkel et al., 2000; Markel’ et al., 1988; Paulus & Geyer, 1993; Paulus et al., 1999; Tanaka et al., 2012). Exploration Efficiency emerged as a third major component of exploration behavior. However, depending on the exploration task, our validation analysis showed the need to further differentiate between goal-directed and free exploration. Lastly, our data suggested that the interpretation of the measures *Flight Turnarounds* and *Minimum Polygon* can be heavily influenced by the respective environment layout.

Contrary to our prediction, we observed that *Path Length* and *Pausing* did not form a Locomotor Activity cluster on their own, but instead were part of the “Exploratory Activity” cluster. This is not only in contrast to the aforementioned animal studies investigating the factor structure of exploration behavior, but also to other animal studies reporting a different reaction to behavioral or pharmacological interventions in measures of locomotor versus exploratory activity (Brudzynski & Krol, 1997; Kakade & Dayan, 2002; Leyland et al., 1976; Minassian et al., 2015; Paulus et al., 1998). One reason that we did not identify a specific locomotor activity cluster could be that animal studies sometimes not only include measures of locomotor activity representing movement across space (i.e., *Path Length* and *Pausing*), but also elements of activity at a single position without spatial movement. For example, this could include rearing (standing on hind legs) or poking behavior (object interaction by poking objects with the head, Paulus & Geyer, 1993), which represent types of behavior that we did not quantify in the present dataset or that may not be relevant in human research. Furthermore, since our dataset describes the exploration of a virtual environment, it is important to keep in mind that recent studies showed that the translation between different presentation methods and between virtual environments and the real world can be challenging (Clemenson et al., 2020; Hejtmanek et al., 2020; but also see Zisch et al., 2022). This might be especially important with respect to the generalization of locomotor and efficiency measures, as movement activity in the current study reflects something qualitatively different from movement activity in real-life studies (i.e., button presses versus more effortful real-life locomotion). Alternatively, the lack of discriminatory power for the *Path Length* and *Pausing* measures in our study could be explained by the length of the observation period in combination with the size of the explored environment. In animal experiments, environments typically are quite small, while at the same time exploration sessions were much longer in the aforementioned animal studies (up to 60 min). In contrast, our virtual environments were comparatively large in relation to the available exploration time. It is therefore possible that the animals in these studies had more time for “idle movement” not directly related to exploration, since full coverage of the area could be achieved more quickly due to the smaller area. On the other hand, for our participants the time was likely too short to show any movement activity not related to exploration. We therefore conclude that if the observation period is relatively short in relation to the size of the to-be-explored environment, measures of locomotor activity like *Path Length* might give an accurate representation of exploratory activity as well. However, for longer periods of exploration, this measure may reflect mere locomotor activity, as observed in the animal literature.

**Summary and future directions**

Overall, our data showed that currently used measures of human exploration behavior describe three core aspects of exploration: the extent of exploration, the spatial shape of the trajectory, and the efficiency of exploration. Crucially, in the case of exploration efficiency, we show that there is a further differentiation into a goal-centered versus a more general, area-centered component. The characterization of these overarching components of exploration behavior further supports more systematic and specific ways to analyze human spatial exploration behavior. By sharing data and code for our analyses, we provide the necessary tools, as well as the opportunity to further cross-validate and generalize the present findings to other datasets and/or populations.

## Supplementary Information

Below is the link to the electronic supplementary material.

Supplementary file1 (DOCX 2375 KB)

## Data Availability

The individual trajectories of both the NEMO as well as the SILCTON study are freely available at https://github.com/valentinbaumann/explorationMeasures*.* For the NEMO virtual environment, please contact Judith Schomaker. For the SILCTON environment, see the project website at https://osf.io/fykr7/.

## Code availability

The code to recreate preprocessing, feature extraction and the statistical analyses is freely available at https://github.com/valentinbaumann/explorationMeasures*.*
