# Supplementary material for: Towards a characterization of human spatial exploration behavior
Source: Behav Res Methods. 2025 Jan 22;57(2):65. doi: 10.3758/s13428-024-02581-3 (PMC11754322; doi:10.3758/s13428-024-02581-3)
Supplement: Supplementary file 1 — Supplementary file1 (DOCX 2375 KB) [file 13428_2024_2581_MOESM1_ESM.docx]

# Supplementary Material for “Towards a characterization of human spatial exploration behavior”

## Section A: Feature Descriptions

### Path Length

*Path Length* represents how much distance a person covers in an environment. It is defined as the sum of all step lengths, with step length calculated as the Euclidian distance between two consecutive points in the trajectory. The value of *Path Length* can vary between zero (no movement at all) and any positive value. Note that this measure cannot discriminate between self-generated movement and passive movement (i.e., falling, sliding, teleportation or any other transportation by external means) and depends on the sampling rate used (Mandelbrot, 1967), movement speed (constant or variable) as well as the measurement dimension (2D or 3D). For previous uses of *Path Length* in human research see (Clemenson et al., 2019; Farran et al., 2022; Meade et al., 2019).

### Pausing

*Pausing* represents how much time an individual spends during exploration without showing any movement activity. It is defined as the sum of all steps where there is no movement multiplied by the respective sampling rate. For a previous application of *Pausing*, see (Gagnon et al., 2016).

### Area Covered

*Area Covered* represents the size of the area covered by an individual. It is defined as the amount of tiles in a grid superimposed on the explored area that were visited at least once. This grid can be represented as a 2d-histogram, where each grid cell represents a histogram bin and takes a value of one if its corresponding area was entered and is zero otherwise (Fig. S1 A). Its value can vary between one (only the starting bin was entered) and any other discrete positive value up to the maximum amount of bins in the histogram. Note that the absolute value of *Area Covered* depends on the bin size, with smaller bin sizes resulting in a higher overall number of bins and a greater *Area Covered* value. For previous applications of *Area Covered* in human research see (Baumann et al., 2020; Farran et al., 2022)

### Roaming Entropy

*Roaming Entropy* (also “location entropy”, see (Särkelä et al., 2009)) represents a measure of the spatial extent and distribution of movement. In addition to measuring the extent of exploration, it also takes the amount of exploration at a given location into account. Conceptually, it is a spatial form of the Shannon Entropy (Weaver, 1953), which represents the amount of information a certain set of data contains, or, framed differently, how much uncertainty is in a dataset. In terms of spatial exploration, *Roaming Entropy* is defined as the sum of the probabilities for a subject *i* being at position *j*, standardized by the log of all possible locations *k*:

$${RE}_{i}=-\sum_{j=1}^{k} \left( p_{i,j}\log p_{i,j} \right)/\log k$$

Similar to *Area Covered*, The available positions are defined using a grid approach, where positions are represented by bins in a 2d-histogram of the area (Fig. S1B). The parameter *k* represents the total number of positions available. This is either a known property of the environment or needs to be estimated in some way (i.e., as the number of bins entered by at least one subject, resulting in a rough approximation of the accessible area). *Roaming Entropy* ranges between zero and one. Values closer to zero indicate a high predictability of where a subject can be encountered at any given time during exploration (a value of zero would indicate a perfect predictability of position, and can only occur if there is no movement at all). Higher values indicate movement that is more spread throughout the available area. A value of one would indicate that each available position was visited with the exact same frequency resulting in equal probabilities across positions. This gives the lowest possible predictability of positions, or, in other words, the greatest uncertainty possible. Note that the predictability purely focuses on the spatial aspect, and does not take into account the temporal order of movement. For example, while intuitively the position of a person moving only straight ahead at constant speed might be perfectly predictable, Roaming Entropy still will have a value > 0, since without the temporal information on the sequence of moves the position cannot be predicted with 100% accuracy. An interactive example of *Roaming Entropy* can be found at <https://www.brandmaier.de/roamingentropy/>.

For previous applications of *Roaming Entropy* in human research see (Brunec et al., 2022; Cen et al., 2022; Clemenson et al., 2019; Heller et al., 2020; Särkelä et al., 2009; Schomaker et al., 2022) . Also, note that there are other entropy measures in the literature, as entropy is able to quantify uncertainty for any kind of data. For example, entropy based on velocity or accelerometer data has been used (Li & Li, 2014; Perry et al., 2009), as well as data combining both spatial position as well as other behavioral events (Paulus et al., 1990) or entropy based on heading direction (Cen et al., 2022).


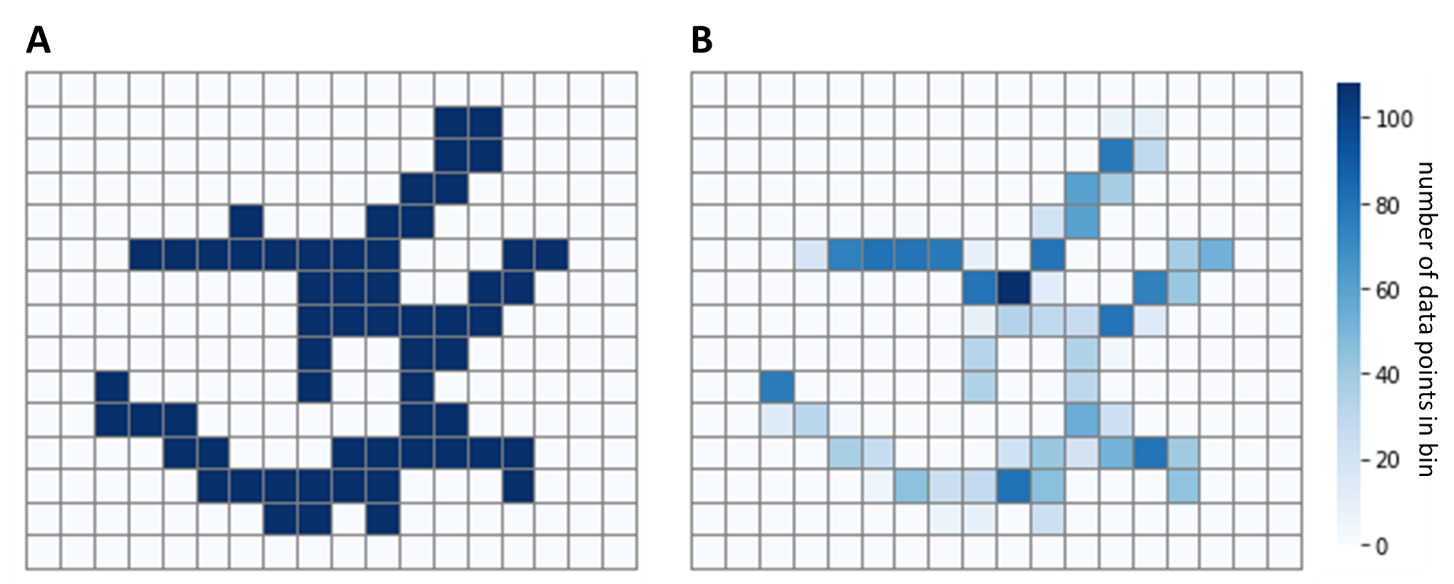


Figure S1. To compute *Area Covered* and *Roaming Entropy*, a grid is mapped on the original trajectory. This results in a 2d histogram where each bin represents a part of the underlying area. For *Area Covered*, a bin takes the value 1 if the corresponding area has been entered during exploration (A, dark blue), and is zero otherwise (A, white). We can also quantify to what extent a bin is explored by counting how many data points fall into a given bin. This frequency map (B) can then be converted to a probability map, from which *Roaming Entropy is computed*.

### Area Efficiency

To assess the efficiency of exploration, previous work in animals proposed an efficiency score, which compared the area explored to the “effort” necessary to cover this area (Rosenberg et al., 2021). However, instead of using only specific landmark t locations as in Rosenberg et al. (2021), we here compare the total area covered to the total amount of movement spent, as this gives a more general estimation that can also be used in environments without defined end points . As this new measure therefore quantifies the efficiency in covering an area, we call it *Area Efficiency* (*Area Efficiency* = *Area Covered* / *Path Length*).

$$Area Efficiency= \frac{Area Covered}{Path Length}$$

Note that this measure may carry a different meaning for exploration in virtual versus real environments, as the perceived effort to initiate and maintain movement may be very different (i.e., pressing a key versus walking).

### Minimum Convex Polygon

In human as well as animal research, the Minimum Convex Polygon has been used to describe the activity space of an individual (Hirsch et al., 2014; Šimon et al., 2019). It is computed by calculating the smallest possible polygon that covers all points of a trajectory (see Fig. S2). We then calculate the area of this polygon to get the size of an individual’s activity space. A greater activity space area would indicate that exploration is very spread out, while a small activity space would indicate that an
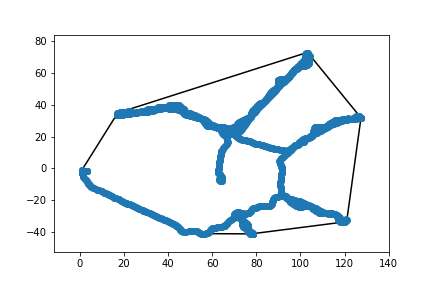
individual explored within a rather compact area.

Fig. S2. The Minimum Complex Polygon describes the smallest possible polygon that encompasses all points of a trajectory.

### Revisiting

*Revisiting* measures how often a participants returns to the same location (Gagnon et al., 2016, 2018; Munion et al., 2019). It is calculated by counting how many data points prior to the current position fall inside a predefined area around the current position (note that while within the area, the points directly preceding the current position are not counted as “revisiting”). This is repeated for all points in the trajectory and averaged to give the *Revisiting* score (Fig. S3). Revisiting previously has been interpreted as an indicator of caution as well as efficiency during exploration, with higher scores representing less efficient and more cautious exploration.


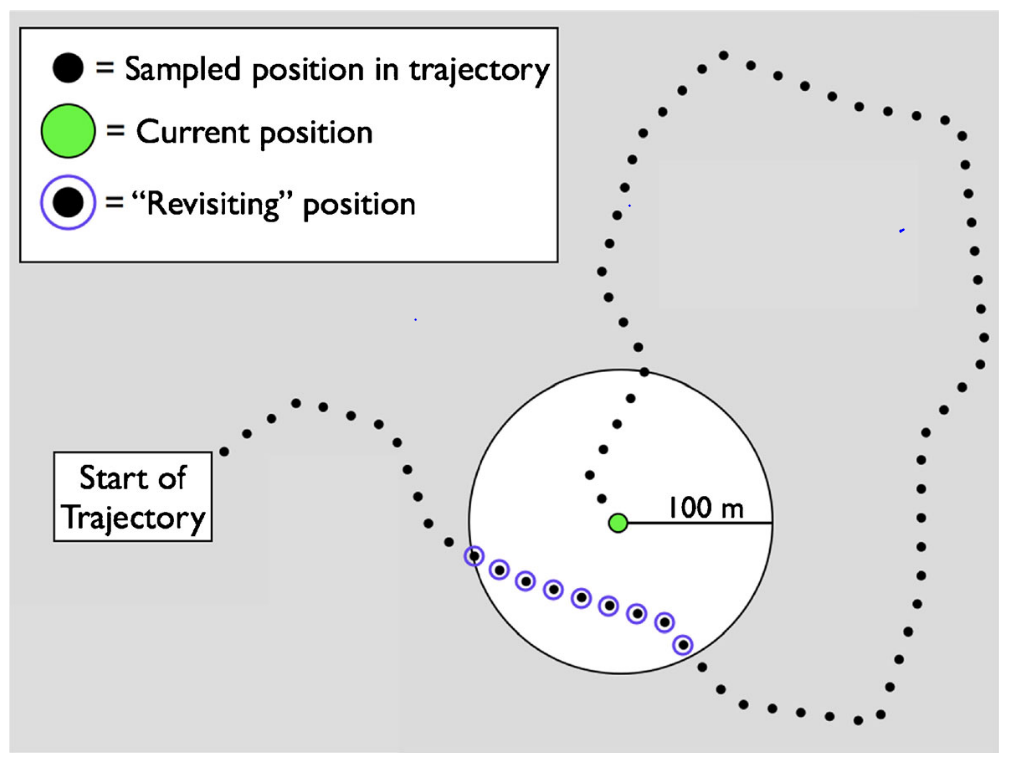


Figure S3. *Revisiting* is calculated by counting how many points prior to the current position fall inside a predefined area around the current position (note that while within the area, the points directly preceding the current position are not counted as “revisiting”). This is repeated for all points in the trajectory and averaged to give the *Revisiting* score. Figure from (Gagnon et al., 2016). Note that *Revisiting* heavily depends on the size of the circle area, with a larger area size resulting in a larger *Revisiting* score.

### Turnarounds (original)

*Turnarounds* measure how often an individual moves in the directly opposite direction and thus retraces his or her own path. It is defined as the number of times that an individual is changing their heading direction by 180 degrees, taking all consecutive data points of the trajectory into account. This requires that heading angles are computed in such a way that they range from 0 (no change in heading direction) to 180 degrees (complete reversal of heading direction) for both left and right turns. Note that the heading direction is computed based on changes in movement, and does not take any differences in the actual viewing angle into account. A previous study used *Turnarounds* as an indicator of exploration efficiency (Farran et al., 2022), with lower values indicating fewer turnarounds and therefore a better pathing efficiency.. However, our data showed that this conceptualization of *Turnarounds* may not accurately capture path retracing and consequently may not represent a measure of exploration efficiency (see main text, Fig. 5).

### Flight Turnarounds

As the original *Turnarounds* measure only captures very short and small scale movement sequences, it might be desirable to modify the measure to represent longer term changes in heading direction (see main text, Fig. 5). We therefore propose to measure heading angles not on the scale of steps (data points represent movement steps at the scale of original sampling frequency), but on the scale of flights. A flight summarizes all steps with a continuous movement direction, given a predetermined degree of deviation (Rhee et al., 2011). Here, we resampled the trajectory to the flight scale using the Ramer Douglas Peucker (RDP) algorithm (see Hirschmann, 2016, for the python implementation). Note that here the value of *Flight Turnarounds* depends on the value of the parameter *ε*, which controls how much the course of the to be estimated flight may deviate from the original data points. For a similar application of flight based turnarounds in human research also see the “rectangular method” presented in (Rhee et al., 2011).

### Landmarks Visited

*Landmarks Visited* represents the total number of unique landmark locations visited, with higher values indicating a greater number of explored landmarks. A landmark “visit” is defined as the participant entering a predefined (in our case circular) area around the landmark. Note that the value of *Landmarks Visited* depends on the size and shape of the area. For an application of *Landmarks Visited* in human research see (De Alencar et al., 2015; Fornasari et al., 2013; Meade et al., 2019; Schomaker et al., 2022).

### Landmark Revisits

*Landmarks Revisits* measures how often Landmarks are visited after their initial discovery and has been previously used as an index of pathing efficiency. It is defined as the number of visits to any landmark, not counting the first visit. A “visit” is defined as the participant entering a predefined (in our case circular) area around the object. Lower values indicate less revisits and therefore better pathing efficiency. For an application of *Landmark Revisits* in human research see (Fornasari et al., 2013).

### Landmark Efficiency

*Landmark Efficiency* measures the efficiency of exploring different landmarks. Similar to the efficiency method proposed in a recent animal study (Rosenberg et al., 2021), it compares the number of landmarks visited to the to the effort needed to get there, represented by the total amount of movement (*Landmark Efficiency* = *Landmarks Visited* / *Path Length*). Note that this measure may carry a different meaning for exploration in virtual versus real environments, as the perceived effort to initiate and maintain movement may be very different (i.e., pressing a key versus walking).

### Fractal Dimension

*Fractal Dimension* is a geometric measure that quantifies the complexity of a line as the ratio of the change in the line’s detail to the change in the scale at which the line is measured (Mandelbrot, 1967). In movement analysis, *Fractal Dimension* has been used to quantify a trajectory’s tortuosity and is usually calculated using the dividers method, where the length of a trajectory is measured for a range of step sizes (the “dividers”), thus looking at the trajectory at different spatial measurement scales (Fig S4 A) (Benhamou, 2004; Nams, 2006; Nams & Bourgeois, 2004). The slope of the linear model fitted to a log-log plot of step sizes and path lengths can then be used to calculate the *Fractal Dimension* of the trajectory (*Fractal Dimension* = 1 – *slope*) (Fig. S4 B). To make the calculation more robust, a corrected dividers methods has been proposed by (Nams, 2006; Nams & Bourgeois, 2004), which entails running the dividers backwards as well as forwards and additionally corrects for a starting point bias. *Fractal Dimension* can take values between 1 and 2. A value of 1 represents movement in a completely straight line, while higher values indicate more tortuous, curvy movement. At a value of 2, it reflects a line that completely covers its plane (Brownian motion) (Benhamou, 2004). The calculation of *Fractal Dimension* depends on which range of step sizes is selected. (Kulatilake et al., 1997) recommended a minimum suitable range of step sizes that starts at least at 0.5% of the whole Path Length and covers step sizes up to at least the step length corresponding to 10 data points. For applications of *Fractal Dimension* in human spatial exploration research see (Kearns et al., 2010; Perry et al., 2009; Yaremych et al., 2019).


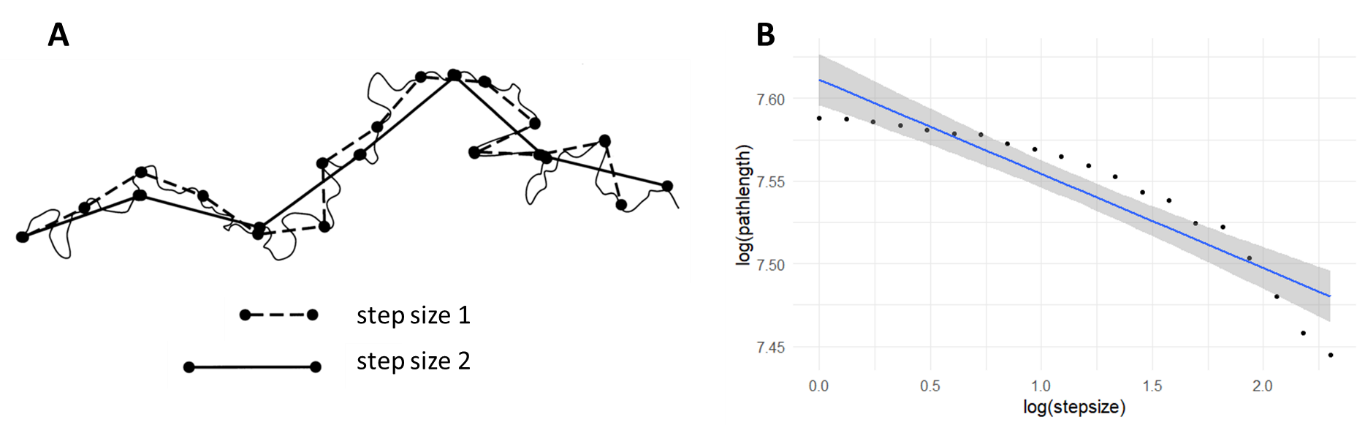


Figure S4. The dividers method assesses the length of the trajectory at different spatial scales represented by the different step sizes (A). *Fractal Dimension* can then be computed from the slope of a linear model of path length and step sizes in a log-log plot (B). Part A adapted from (Gonzato, 1998).

### Sinuosity

*Sinuosity* is another a measure of path tortuosity (Benhamou, 2004; Bovet & Benhamou, 1988). It is based on the turning angles of the trajectory (Fig. S5) as well as the distribution of step lengths. The original *Sinuosity* calculation is only valid for trajectories with regular step lengths and is defined as

$$S_{original}=1.18* \frac{\sigma_{q}}{\sqrt{q}}$$

where $\sigma_{q}$ is the standard deviation of turning angles and $q$ the constant step length. If the trajectory forms a straight line, $\sigma_{q}$ is zero and the *Sinuosity* equation also evaluates to zero. If the *Sinuosity* of a trajectory with irregular step lengths is to be computed, the trajectory either has to be re-discretized to a regular step length or the generalized *Sinuosity* formula (Benhamou, 2004) has to be used:

$$S_{generalized}=2\left[ p\left( \frac{1+c}{1-c}+b^{2} \right) \right]^{-0.5}$$

Here, *p* is the mean step length, *c* the mean cosine of turning angles (in radians) and *b* the coefficient of variation of step length. The mean cosine of turning angles is small for high turning angles and high for small turning angles. The straighter a line, the closer the term $\frac{1 + c}{1 - c}$ approaches infinity. For a completely straight line where $\frac{1 + c}{1 - c}=inf$, the equation evaluates to zero. Additionally, the generalized *Sinuosity* is higher for trajectories with smaller and less variable step lengths (expressed through *p* and *b*).


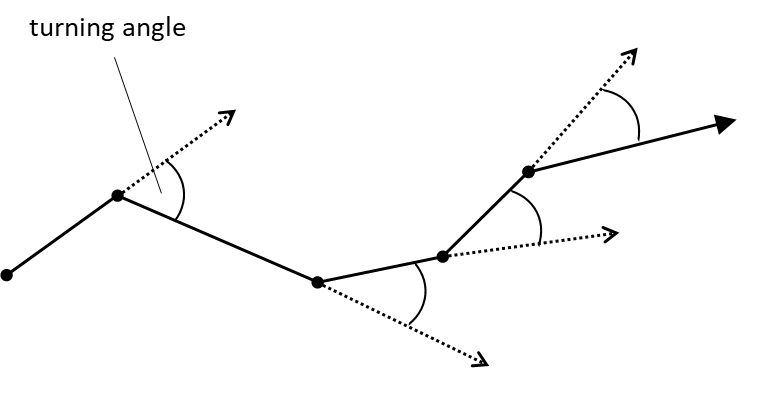


Figure S5: Turning angles across a trajectory (bold line represents the original trajectory, dashed lines the assumed heading direction at each point)

## Section B: Sample Description (NEMO)

A total of 487 visitors of the NEMO Science Center in Amsterdam age range 8 to 77 years) volunteered to participate in this study, from which we included 409 participants in the final sample (Fig. S6, see main text for exclusion criteria). All participants or a participant’s parent in case of minors, gave written informed consent. Participants could choose to perform the tasks in Dutch or English. The study was approved by the Psychology Research Ethics Committee of Leiden University, the Netherlands. All procedures were in line with the Declaration of Helsinki (1964, and later amendments), and adhered to the relevant COVID-19 guidelines and regulations at that time.


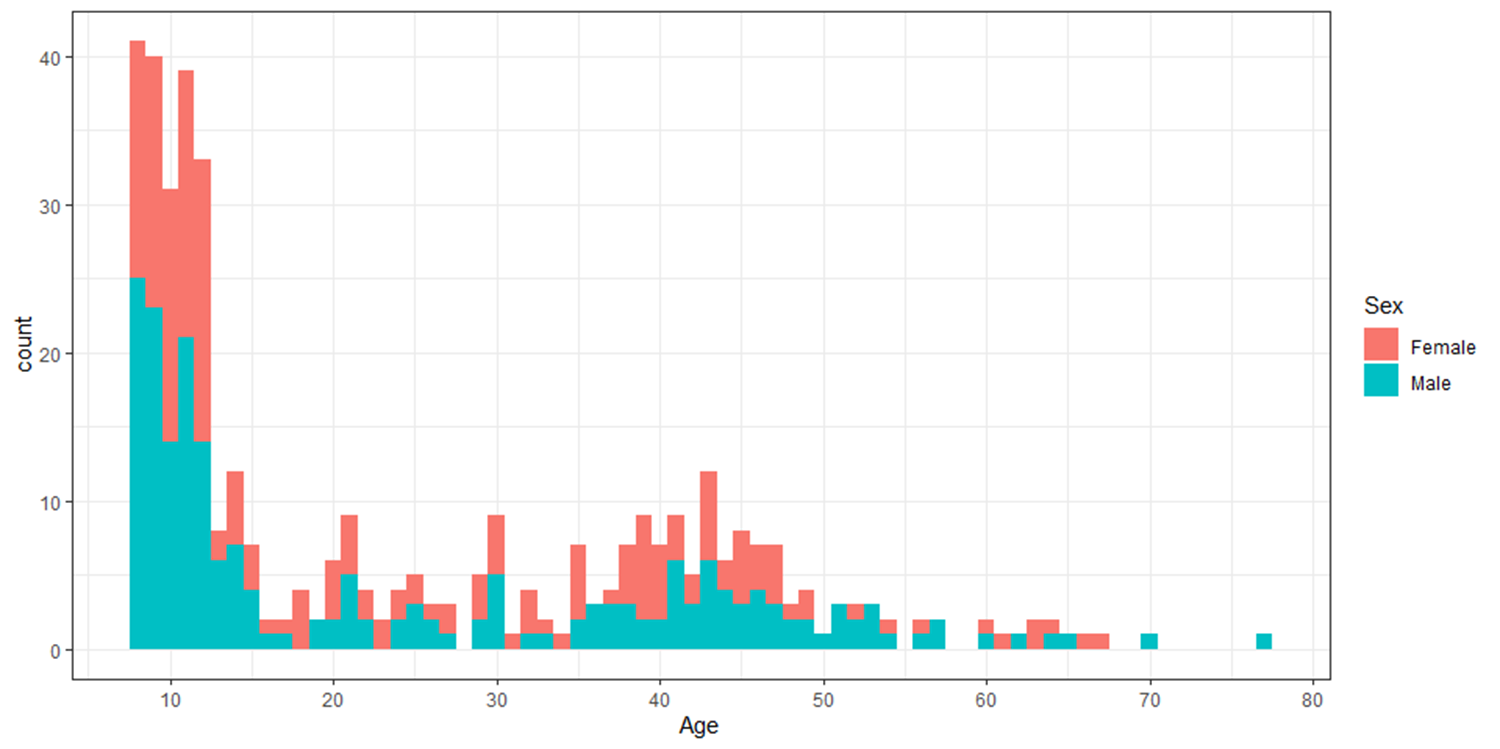


Figure S6. Distribution of age and gender across the sample.

## Section C: Experiment Description (NEMO)

### Stimuli and apparatus

The virtual environments were created using Unity Version 2017.2.21f1 (Unity Technologies, 2017). The environments were matched in size, path length, and number of intersections. Both environments consisted of fantasy islands with unusual landmarks (such as a slot machine) at intersections or road endpoints, including land and a body of water. Participants could move using the WASD keys on the keyboard and used the mouse to determine the heading direction. During exploration the X, Y and Z coordinates of the moving agent were logged for all time points with a sampling rate of about 15 Hz. Landmarks were objects from the Unity Asset store, and included a wide range of easily recognizable objects, such as an airplane and desk chair. Memory of landmarks was assessed later in a landmark recognition task (for results, see (Schomaker et al., 2022)). The landmark task and the novelty seeking questionnaire were presented using E-Prime 3.0 software (Psychology Software Tools, Pittsburgh, PA).

### Procedure

Before participation, participants (or in case of underaged participants, their parents) read the information letter and were given the opportunity to ask questions. After giving written informed consent, the participants were seated before they performed a series of tasks on a laptop. Participants received scripted verbal instructions regarding how to navigate through the environment. The space bar could be used to jump, although there was no function in jumping, as one could not jump on top of things. Participants were instructed that they could navigate freely but should try to stay on the paths (note that it was nevertheless possible to also explore the area off the paths). During the first *familiarization phase*, participants explored the environment for 150s. After exploration, they were asked to indicate their happiness (“How happy are you?”, from 1 = extremely unhappy to 9 = extremely happy) and arousal (“How aroused are you?”, from 1 = very calm to 9 = very excited) on a *visual rating scale* with Self-Assessment Manikins. They could use the number keys to indicate their answers, and completing the ratings took less than 1 minute. During the following *second exploration phase* participants explored either the same (i.e., familiar) or a new environment for another 150 s (i.e., novelty and environments were counterbalanced). After this exploration, participants were asked to rate their happiness and arousal levels again on the same two rating scales as before the first exploration. Note that we only analyzed data from the first exploration session here.

In the remainder of the experiment, participants performed a word learning task and a visuomotor adaptation task. Details on these tasks as well as the results have been reported in Schomaker et al. (2022) and Ruitenberg et al. (2022), respectively. Finally, participants provided demographical information and completed a questionnaire that assessed their novelty seeking personality trait. Adults (>17) completed the novelty seeking scale of the Tridimensional Personality Questionnaire (Cloninger et al., 1991), whereas children and adolescents filled out a simplified and abbreviated (20 item) version of the questionnaire (note that this is why we did not include data of children and adolescents in the novelty seeking analysis). Each question remained on the screen until a response was given. All questions could be answered in about 2-5 minutes. Afterwards feedback was shown on basis of the total NS score (i.e., with a subdivision into low, medium, and high scorers). These cut-off scores were only used to provide the participants feedback and were not used in any analyses.


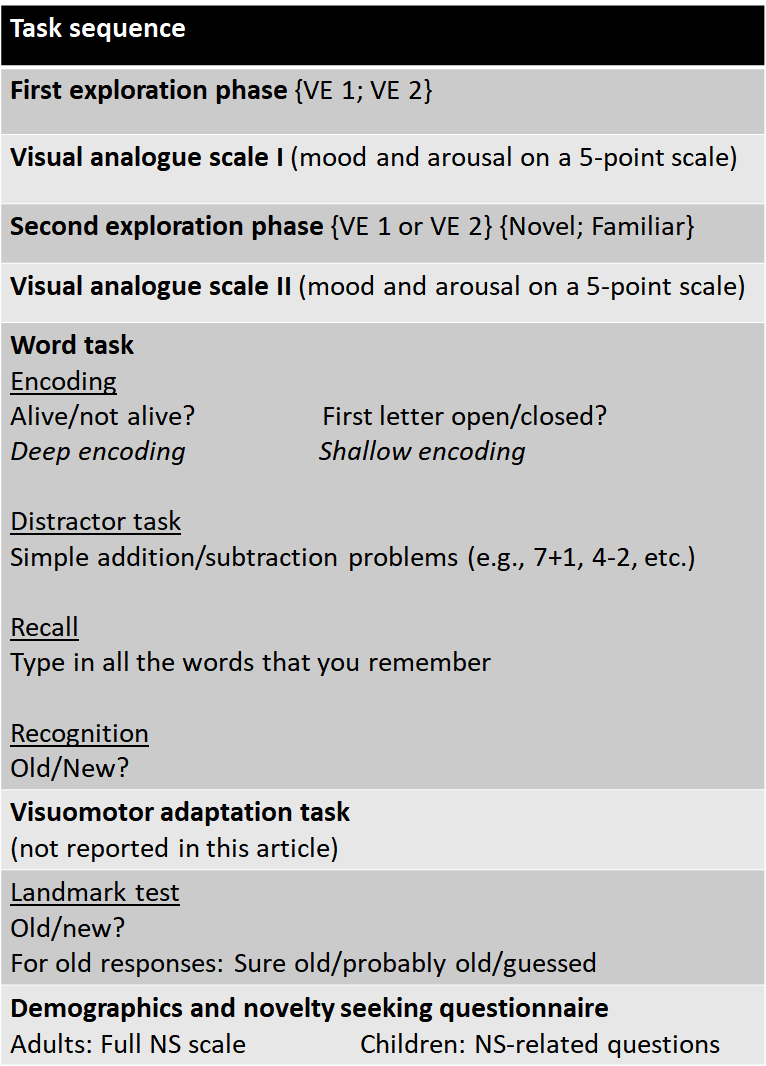


Figure S7. Experimental task sequence. Tasks are shown in sequential order from top to bottom. Note that In this article, we only used data from the first exploration phase and the novelty seeking questionnaire data (from Schomaker et al., 2022)).

## Section D: Parameter Selection

To evaluate the influence of individual parameter choices, we followed a two-step procedure. First, we selected an initial starting value, preferably based on either previous literature or specific environment characteristics (e.g., the distance travelled in a certain amount of time, or distances between landmarks) to get an externally valid estimation. Second, we evaluated this initial parameter by iterating across a range of lower as well as larger parameter values (going from ¼ the starting value to 4x the starting value). With the resulting data, we then used descriptive statistics to see how changes of the parameter value changed the parameter distribution in terms of variability, skewness, bottom/ceiling effects, and kurtosis. Additionally, we plotted the correlations of the different parameter values to check how the different parameter values related to each other. If there was a large variation between the different parameter distributions, we chose the parameter with the highest variability, smallest skewness, smallest bottom/ceiling effects, and smallest kurtosis. In the following, we describe the decision process for each of the investigated exploration measures.

For *Area Covered* and *Roaming Entropy*, we used an initial bin size representing an area of 14x14 virtual meters (vm), which was equivalent to the median distance moved in three seconds (equal for both environments). Using a bin size of 14, we then iterated across a parameter range of 3,7,14,28 and 56 vm. As our data did not suggest any extreme changes between parameter values, we therefore chose 14 as the final parameter value (Fig. S8 and S9, table S1 and S2).

For *Landmark Visits* and *Landmark Revisits*, we chose an initial radius of *r* = 20 vm centered around that landmark as a starting value. This radius was chosen, since it roughly corresponded to the distance at which a landmark would be clearly visible. The iteration of the different parameter values showed that with a radius of 20 vm values variability was quite skewed, as the majority of participants scored all landmarks as “visited” (Fig. S10, table S3). We therefore chose a value of 10 vm for our analysis, which produced much more evenly distributed parameter values. To keep things equal between related measures, we applied the same radius for *Landmark Revisits* (Fig. S11, table S4).

For *Revisiting*, we used an initial radius of *r* = 14 vm, similar to the bin size in *Area Covered*. The analysis of parameter values showed, that at small and medium radii, there seemed to be a bottom effect, with a majority of cases at the lower end (Fig. S12, table S5). This shifted to a less skewed distribution at very high parameter values. This is likely due to high radii naturally producing more Revisits. However, in this case, this might not be desired, as at very high radii even very different areas on the map fall into the same Revisiting radius. We therefore decided to stick with the initial value of 14 vm.

For *Fractal Dimension*, we followed recommendations from the *trajr* package (McLean & Skowron Volponi, 2018) as well as from Kulatilake et al. (1997) and specified an initial range of step sizes from which the fractal dimension was estimated based on the median step length across all trajectories. We chose 20 different step lengths, starting at half the median step length and going up to 10 times the median step length, with the step lengths selected to give equal distances in the log-log space (procedure as suggested in the documentation of the *trajr* package, McLean & Skowron Volponi, 2018). During parameter iteration, we varied the range of step lengths, going from ¼ the initial values up to 4x the initial values. As our data showed only minor variation in the data, we decided to stick with the initial range of step lengths.< (Fig. S13, table S6)

### Area Covered

Parameter iterated: *bin size*; parameter range: *[3,7,14,28,56]*; initial parameter value: *14*; final parameter value: *14*


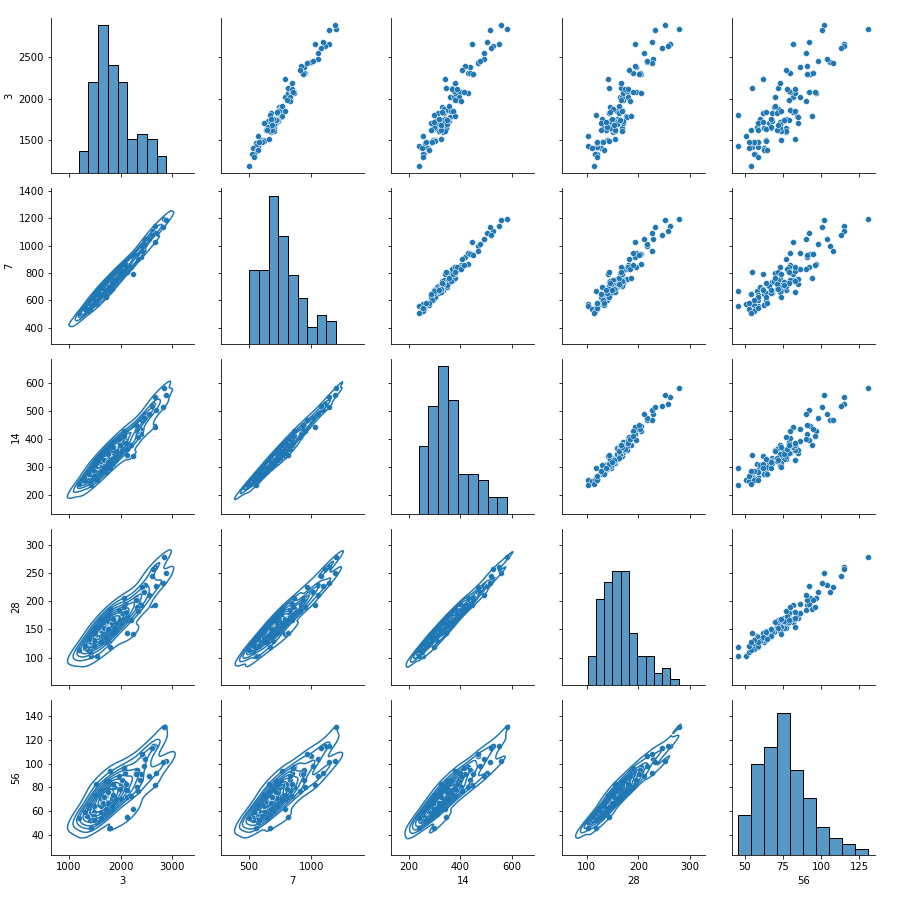


Figure S8. Distributions of the respective outputs of the *Area Covered* computation using five different parameter values (diagonal) as well as the correlogram of the relationship between the different outcomes (lower half: density plot, upper half: scatterplot).

Table S1. Area Covered

|  | coefficient of variation | skewness | kurtosis | percent cases in lowest 10% of values | percent cases in highest 10% of values |
| --- | --- | --- | --- | --- | --- |
| 3 | 0.204 | 0.711 | -0.139 | 3.883 | 2.913 |
| 7 | 0.21 | 0.825 | 0.201 | 10.68 | 3.883 |
| **14** | **0.214** | **0.877** | **0.419** | **10.68** | **2.913** |
| 28 | 0.219 | 0.877 | 0.738 | 6.796 | 1.942 |
| 56 | 0.216 | 0.727 | 0.603 | 6.796 | 0.971 |

### Roaming Entropy

Parameter iterated: *bin size*; parameter range: *[3,7,14,28,56]*; initial parameter value: *14*; final parameter value: *14*


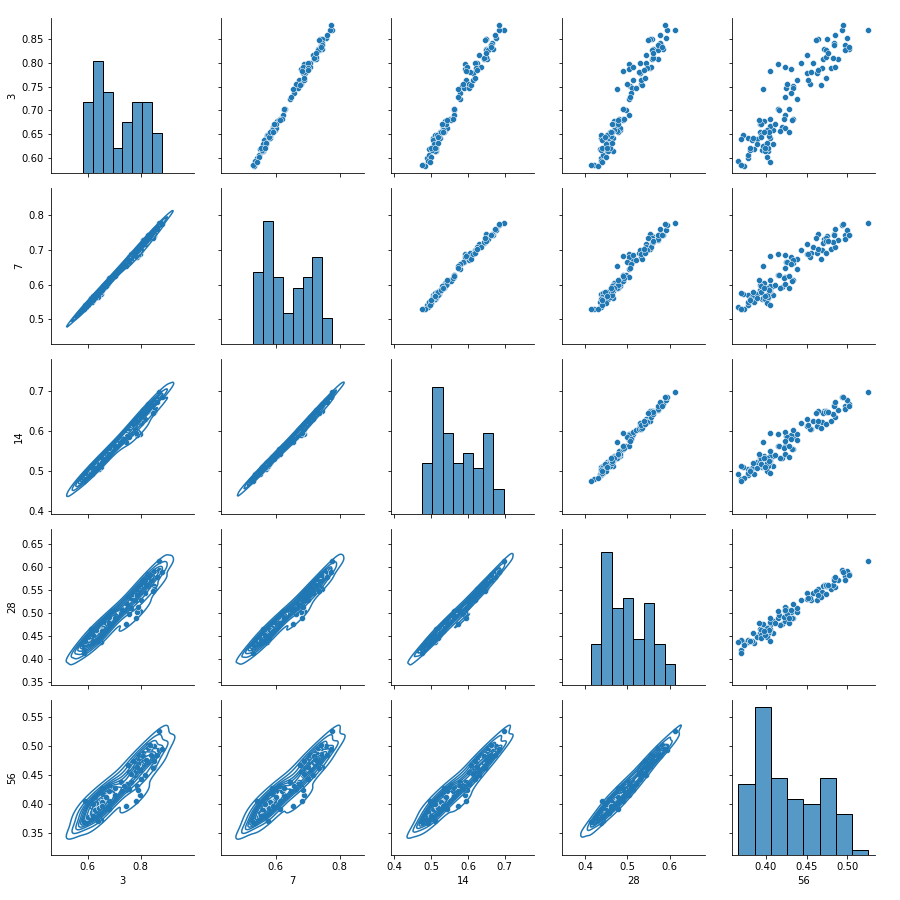


Figure S9. Distributions of the respective outputs of the *Roaming Entropy* computation using five different parameter values (diagonal) as well as the correlogram of the relationship between the different outcomes (lower half: density plot, upper half: scatterplot).

Table S2. Roaming Entropy

|  | coefficient of variation | skewness | kurtosis | percent cases in lowest 10% of values | percent cases in highest 10% of values |
| --- | --- | --- | --- | --- | --- |
| 3 | 0.121 | 0.222 | -1.353 | 7.767 | 5.825 |
| 7 | 0.113 | 0.255 | -1.311 | 8.738 | 4.854 |
| 14 | 0.106 | 0.326 | -1.201 | 7.767 | 3.883 |
| 28 | 0.1 | 0.388 | -1.031 | 2.913 | 1.942 |
| 56 | 0.092 | 0.45 | -0.898 | 10.68 | 0.971 |

### Landmarks Visited

Parameter iterated: *maxDistance*; parameter range: *[5,10,20,40,80]*; initial parameter value: *20*; final parameter value: *10*
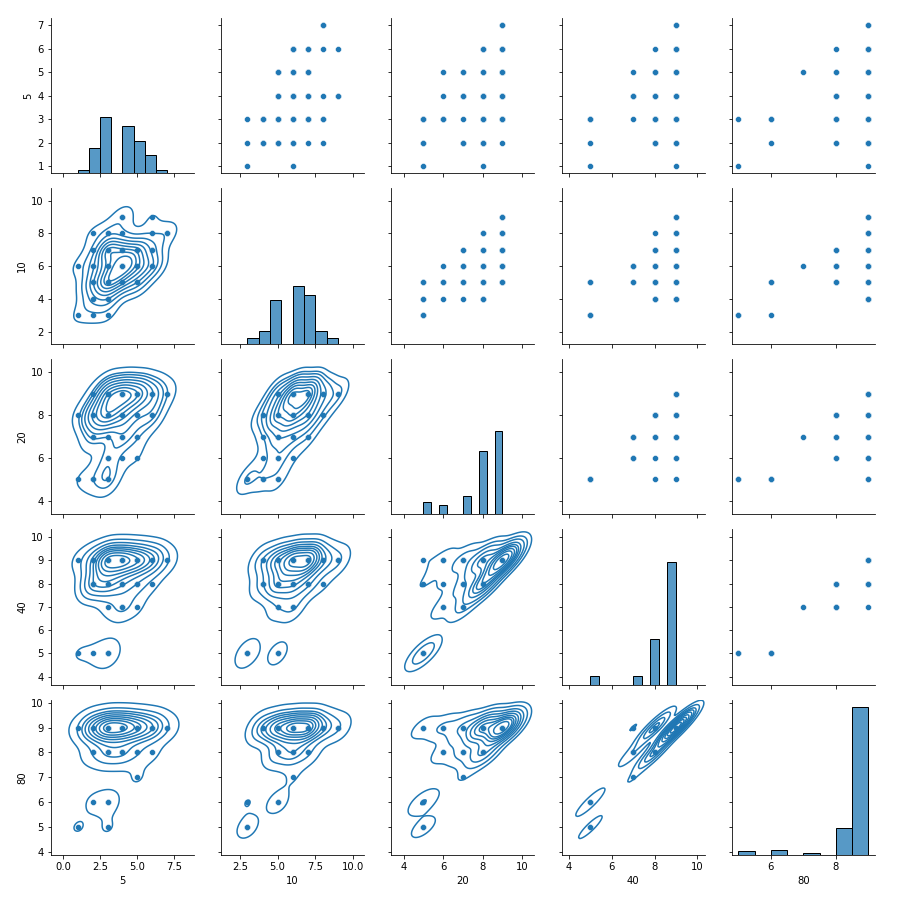


Figure S10. Distributions of the respective outputs of the *Landmarks Visited* computation using five different parameter values (diagonal) as well as the correlogram of the relationship between the different outcomes (lower half: density plot, upper half: scatterplot).

Table S3. Landmarks Visited

|  | coefficient of variation | skewness | kurtosis | percent cases in lowest 10% of values | percent cases in highest 10% of values |
| --- | --- | --- | --- | --- | --- |
| 5 | 0.347 | 0.28 | -0.441 | 1.942 | 1.942 |
| **10** | **0.208** | **-0.056** | **0.091** | **2.913** | **2.913** |
| 20 | 0.145 | -1.344 | 1.081 | 6.796 | 44.66 |
| 40 | 0.114 | -2.38 | 5.798 | 4.854 | 66.019 |
| 80 | 0.093 | -3.061 | 9.695 | 1.942 | 79.612 |

### Landmark Visits

Parameter iterated: *maxDistance*; parameter range: *[5,10,20,40,80]*; initial parameter value: *20*; final parameter value: *10*


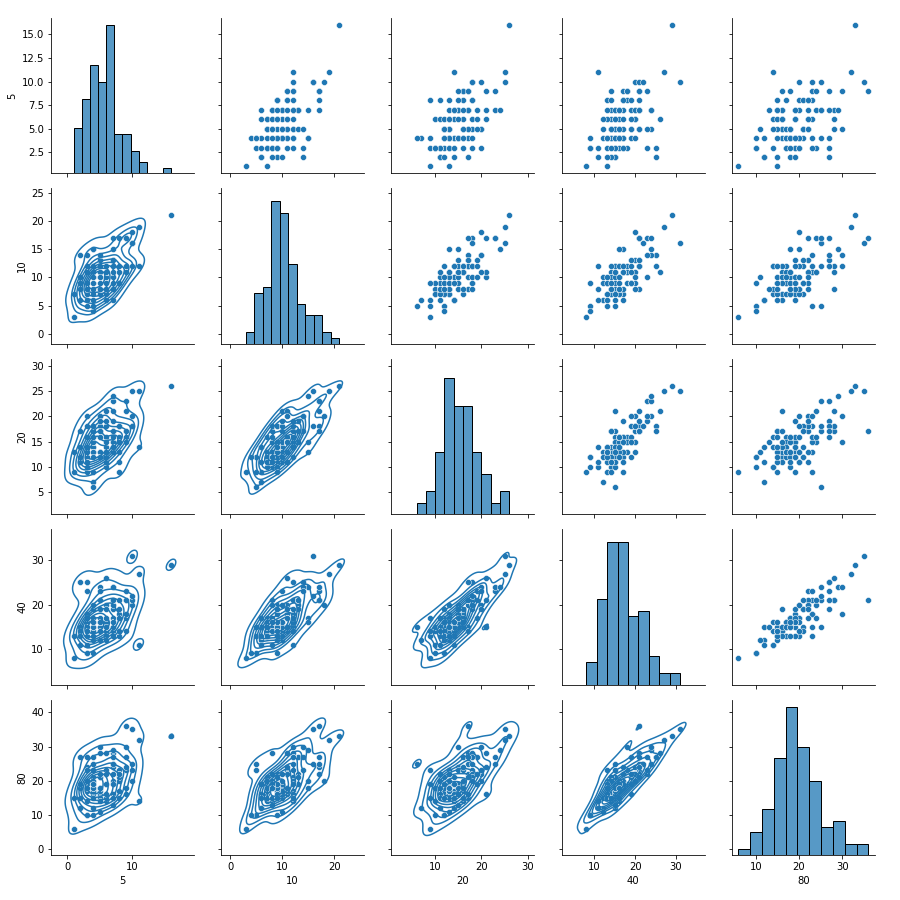


Figure S11. Distributions of the respective outputs of the *Landmark Visits* computation using five different parameter values (diagonal) as well as the correlogram of the relationship between the different outcomes (lower half: density plot, upper half: scatterplot).

Table S4. Landmark Visits.

|  | coefficient of variation | skewness | kurtosis | percent cases in lowest 10% of values | percent cases in highest 10% of values |
| --- | --- | --- | --- | --- | --- |
| 5 | 0.454 | 0.857 | 1.582 | 7.767 | 0.971 |
| **10** | **0.331** | **0.631** | **0.425** | **1.942** | **0.971** |
| 20 | 0.26 | 0.533 | 0.39 | 1.942 | 3.883 |
| 40 | 0.255 | 0.67 | 0.613 | 3.883 | 1.942 |
| 80 | 0.281 | 0.526 | 0.482 | 0.971 | 2.913 |

### Revisiting

Parameter iterated: *radius*; parameter range: *[3,7,14,28,56]*; initial parameter value: *14;* final parameter value: *14*


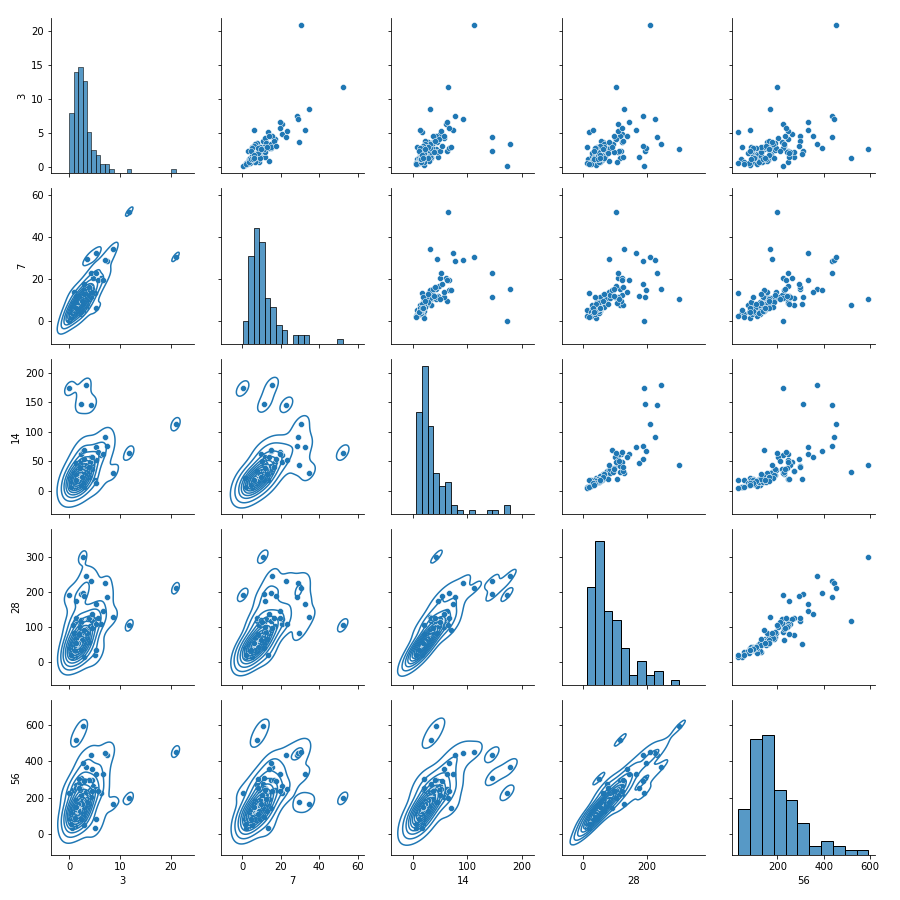


Figure S12. Distributions of the respective outputs of the *Revisiting* computation using five different parameter values (diagonal) as well as the correlogram of the relationship between the different outcomes (lower half: density plot, upper half: scatterplot).

Table S5. Revisiting

|  | coefficient of variation | skewness | kurtosis | percent cases in lowest 10% of values | percent cases in highest 10% of values |
| --- | --- | --- | --- | --- | --- |
| 3 | 0.912 | 3.907 | 23.045 | 41.748 | 0.971 |
| 7 | 0.727 | 2.184 | 6.943 | 21.359 | 0.971 |
| **14** | **0.904** | **2.642** | **8.028** | **45.631** | **1.942** |
| 28 | 0.689 | 1.485 | 2.15 | 23.301 | 0.971 |
| 56 | 0.56 | 1.319 | 2.062 | 12.621 | 0.971 |

### Fractal Dimension

Parameter iterated: *stepSizes*; parameter range: *[1/4* median step size, 1/2 median step size, 1 * median step size, 2 * median step size, 4 * median step size]*; initial parameter value: *1 * median step size;* starting final parameter value: *1 * median step size*


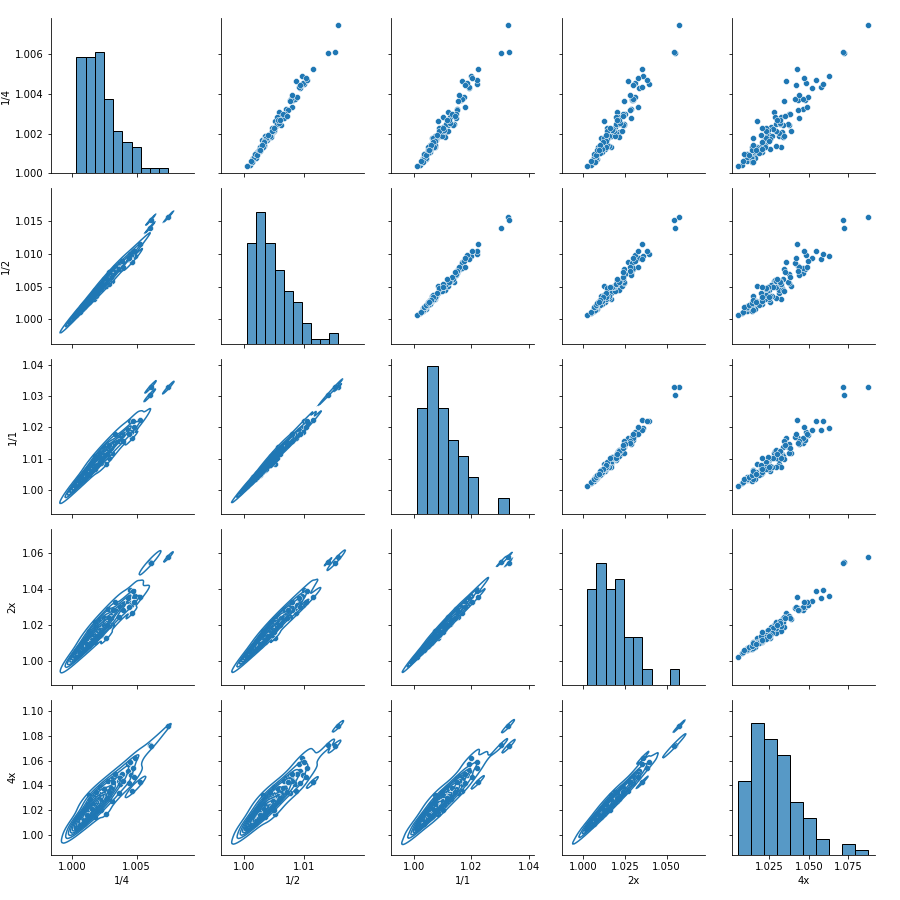


Figure S13. Distributions of the respective outputs of the *Fractal Dimension* computation using five different parameter values (diagonal) as well as the correlogram of the relationship between the different outcomes (lower half: density plot, upper half: scatterplot).

Table S6. Fractal Dimension

|  | coefficient of variation | skewness | kurtosis | percent cases in lowest 10% of values | percent cases in highest 10% of values |
| --- | --- | --- | --- | --- | --- |
| 1/4 | 0.001 | 1.139 | 1.224 | 21.359 | 0.971 |
| 1/2 | 0.003 | 1.167 | 1.432 | 18.447 | 1.942 |
| **1/1** | **0.006** | **1.261** | **1.965** | **19.417** | **2.913** |
| 2x | 0.011 | 1.223 | 1.898 | 17.476 | 2.913 |
| 4x | 0.015 | 1.11 | 1.586 | 13.592 | 0.971 |

## Section E: NEMO clustering for young and old participants


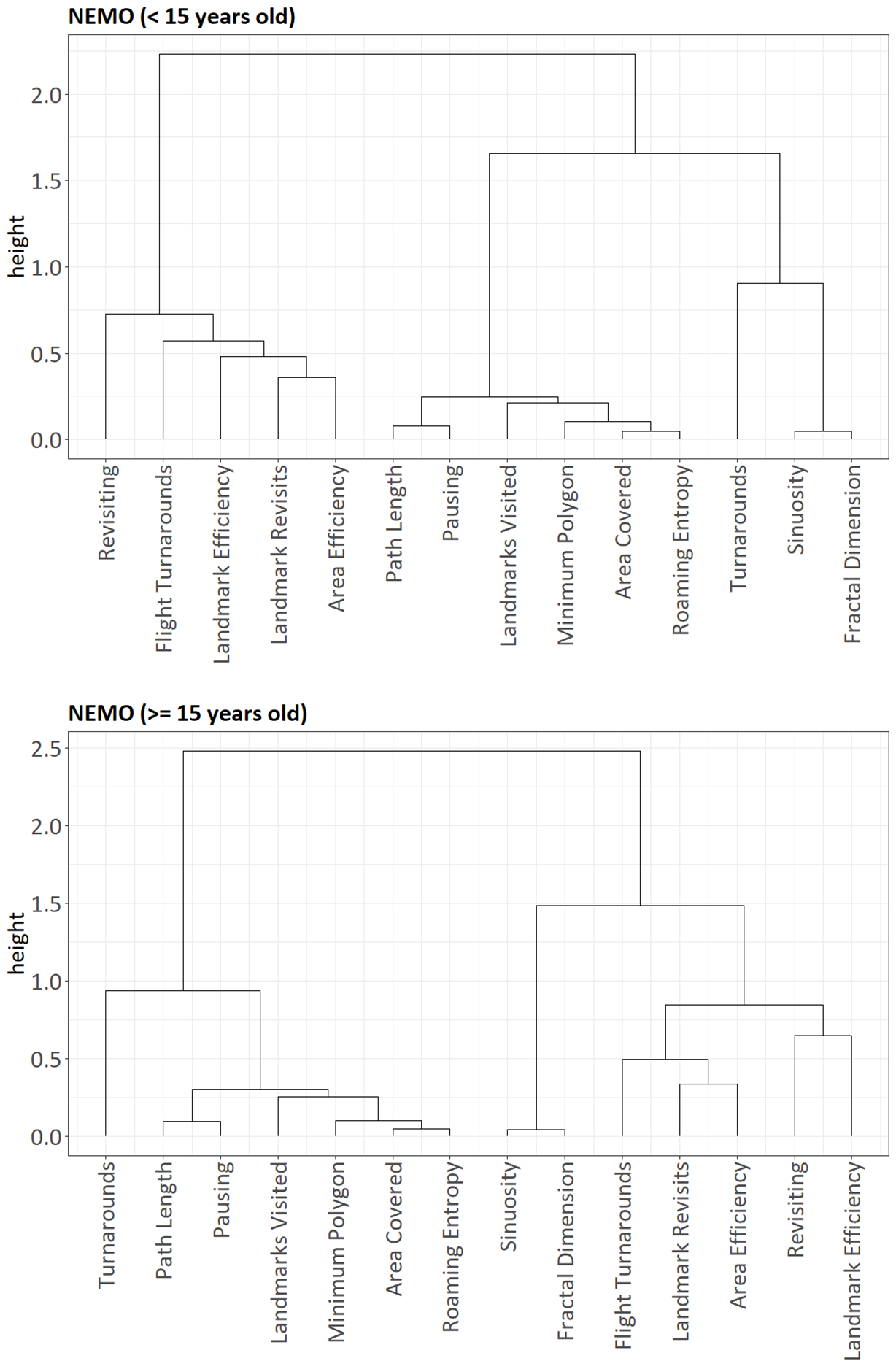


Fig. S14. Clustering results for two subsamples of the NEMO dataset (younger participants: < 15 years, older participants: >= 15 years). Notably, the cluster structure appears to be quite similar for younger vs. older participants.

## Section F: SILCTON clustering for Experiment 1 and 2


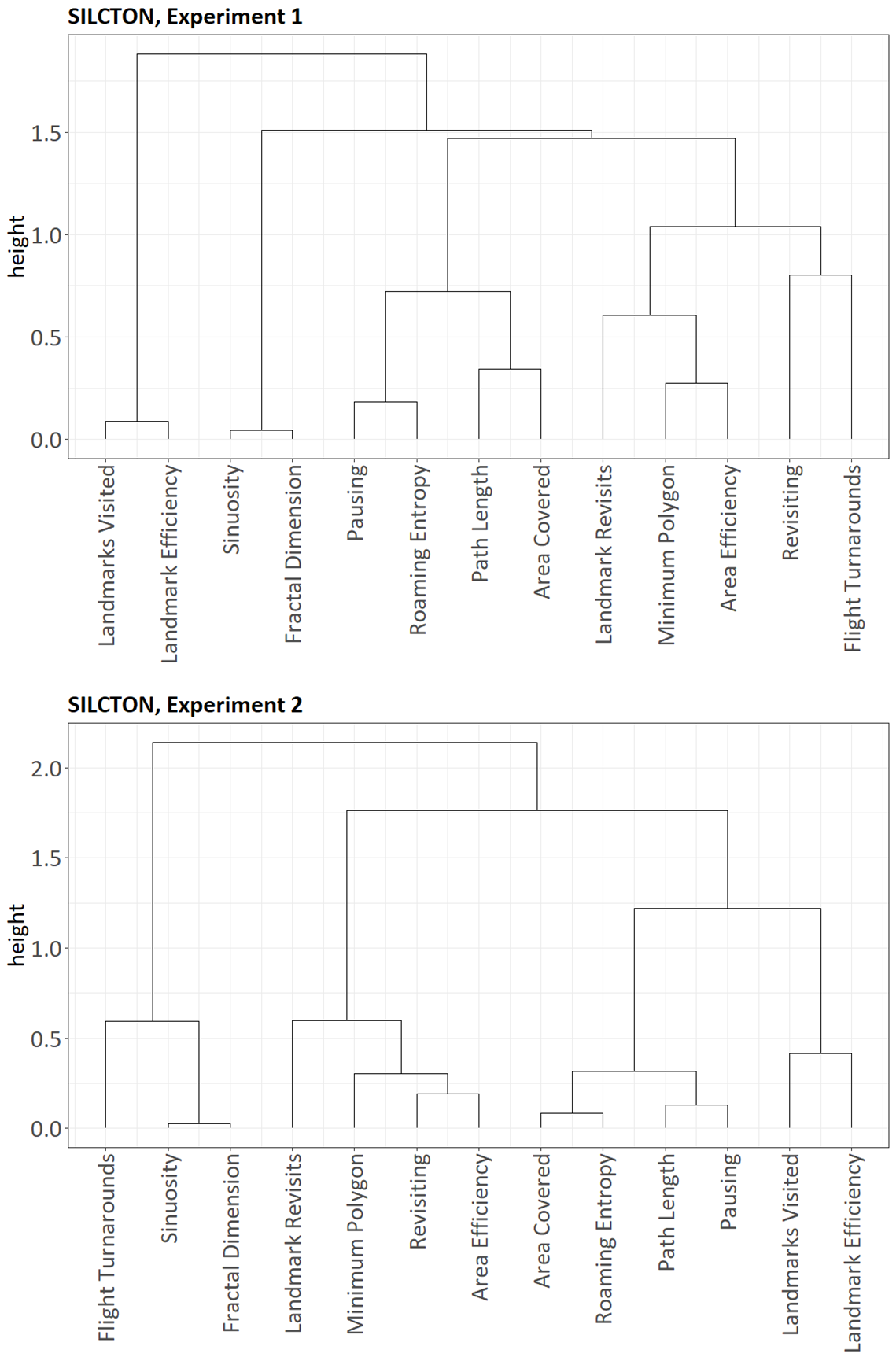


Figure S15. Clustering results for those participants in the SILCTON dataset who explored for 16 minutes (experiment 1) vs. those who explored for 25 minutes (experiment 2).

## Literature

Baumann, V., Birnbaum, T., Breitling-Ziegler, C., Tegelbeckers, J., Dambacher, J., Edelmann, E., Bergado-Acosta, J. R., Flechtner, H.-H., & Krauel, K. (2020). Exploration of a novel virtual environment improves memory consolidation in ADHD. *Scientific Reports*, *10*(1), 21453. https://doi.org/10.1038/s41598-020-78222-4

Benhamou, S. (2004). How to reliably estimate the tortuosity of an animal’s path:: straightness, sinuosity, or fractal dimension? *Journal of Theoretical Biology*, *229*(2), 209–220. https://doi.org/10.1016/J.JTBI.2004.03.016

Bovet, P., & Benhamou, S. (1988). Spatial analysis of animals’ movements using a correlated random walk model. *Journal of Theoretical Biology*, *131*(4), 419–433. https://doi.org/10.1016/S0022-5193(88)80038-9

Brunec, I. K., Nantais, M. M., Sutton, J. E., Epstein, R. A., & Newcombe, N. S. (2022). Exploration patterns shape cognitive map learning. *OSF*, 1–29. https://osf.io/azsgj/

Cen, D., Hodgetts, C. J., & Gruber, M. (2022). States of curiosity and interest shape different aspects of human spatial exploration. *PsyArXiv*. https://doi.org/10.31234/OSF.IO/3GVSK

Clemenson, G. D., Henningfield, C. M., & Stark, C. E. L. (2019). Improving Hippocampal Memory Through the Experience of a Rich Minecraft Environment. *Frontiers in Behavioral Neuroscience*, *13*, 57. https://doi.org/10.3389/fnbeh.2019.00057

De Alencar, L. A., Alvares, L. O., Bogorny, V., Renso, C., & Raffaeta, A. (2015). A rule-based method for discovering trajectory profiles. *Proceedings of the International Conference on Software Engineering and Knowledge Engineering, SEKE*, *2015*-*Janua*, 244–249. https://doi.org/10.18293/SEKE2015-143

Farran, E. K., Blades, M., Hudson, K. D., Sockeel, P., & Courbois, Y. (2022). Spatial exploration strategies in childhood; exploration behaviours are predictive of navigation success. *Cognitive Development*, *61*, 101153. https://doi.org/10.1016/J.COGDEV.2022.101153

Fornasari, L., Chittaro, L., Ieronutti, L., Cottini, L., Dassi, S., Cremaschi, S., Molteni, M., Fabbro, F., & Brambilla, P. (2013). Navigation and exploration of an urban virtual environment by children with autism spectrum disorder compared to children with typical development. *Research in Autism Spectrum Disorders*, *7*(8), 956–965. https://doi.org/10.1016/J.RASD.2013.04.007

Gagnon, K. T., Cashdan, E. A., Stefanucci, J. K., & Creem-Regehr, S. H. (2016). Sex Differences in Exploration Behavior and the Relationship to Harm Avoidance. *Human Nature*, *27*(1), 82–97. https://doi.org/10.1007/s12110-015-9248-1

Gagnon, K. T., Thomas, B. J., Munion, A., Creem-Regehr, S. H., Cashdan, E. A., & Stefanucci, J. K. (2018). Not all those who wander are lost: Spatial exploration patterns and their relationship to gender and spatial memory. *Cognition*, *180*(August 2017), 108–117. https://doi.org/10.1016/j.cognition.2018.06.020

Gonzato, G. (1998). A practical implementation of the box counting algorithm. *Computers and Geosciences*, *24*(1), 95–100. https://doi.org/10.1016/S0098-3004(97)00137-4

Heller, A. S., Shi, T. C., Ezie, C. E. C., Reneau, T. R., Baez, L. M., Gibbons, C. J., & Hartley, C. A. (2020). Association between real-world experiential diversity and positive affect relates to hippocampal–striatal functional connectivity. *Nature Neuroscience 2020 23:7*, *23*(7), 800–804. https://doi.org/10.1038/s41593-020-0636-4

Hirsch, J. A., Winters, M., Clarke, P., & McKay, H. (2014). Generating GPS activity spaces that shed light upon the mobility habits of older adults: a descriptive analysis. *International Journal of Health Geographics*, *13*(1), 51. https://doi.org/10.1186/1476-072X-13-51

Hirschmann, F. (2016). *rdp: Python implementation of the Ramer-Douglas-Peucker algorithm* (0.8). pypi.python.org/pypi/rdp

Kearns, W. D., Nams, V. O., & Fozard, J. L. (2010). Tortuosity in movement paths is related to cognitive impairment Wireless Fractal Estimation in assisted living facility residents. *Methods of Information in Medicine*, *49*(6), 592–598. https://doi.org/10.3414/ME09-01-0079/ID/JR0079-23

Kulatilake, P. H. S. W., Urn, J., & Pan, G. (1997). Requirements for Accurate Estimation of Fractal Parameters for Self-Affine Roughness Profiles Using the Line Scaling Method. In *Rock Mechanics and Rock Engineering* (Vol. 30, Issue 4). Springer-Verlag.

Li, X., & Li, X. (2014). Using Complexity Measures of Movement for Automatically Detecting Movement Types of Unknown GPS Trajectories. *American Journal of Geographic Information System*, *3*(2), 63–74. https://doi.org/10.5923/j.ajgis.20140302.01

Mandelbrot, B. (1967). How Long Is the Coast of Britain? Statistical Self-Similarity and Fractional Dimension. *Science*, *156*(3775), 636–638. https://doi.org/10.1126/SCIENCE.156.3775.636

Meade, M., Meade, J., Sauzeon, H., & Fernandes, M. (2019). Active Navigation in Virtual Environments Benefits Spatial Memory in Older Adults. *Brain Sciences*, *9*(3), 47. https://doi.org/10.3390/brainsci9030047

Munion, A. K., Stefanucci, J. K., Rovira, E., Squire, P., & Hendricks, M. (2019). Gender differences in spatial navigation: Characterizing wayfinding behaviors. *Psychonomic Bulletin & Review*, *26*(6), 1933–1940. https://doi.org/10.3758/s13423-019-01659-w

Nams, V. O. (2006). Improving accuracy and precision in estimating fractal dimension of animal movement paths. *Acta Biotheoretica*, *54*(1), 1–11. https://doi.org/10.1007/s10441-006-5954-8

Nams, V. O., & Bourgeois, M. (2004). Fractal analysis measures habitat use at different spatial scales: an example with American marten. *Canadian Journal of Zoology*, *82*(11), 1738–1747. https://doi.org/10.1139/z04-167

Paulus, M. P., Geyer, M. A., Gold, L. H., & Mandell, A. J. (1990). Application of entropy measures derived from the ergodic theory of dynamical systems to rat locomotor behavior. *Proceedings of the National Academy of Sciences*, *87*(2), 723–727. https://doi.org/10.1073/PNAS.87.2.723

Perry, W., Minassian, A., Paulus, M. P., Young, J. W., Kincaid, M. J., Ferguson, E. J., Henry, B. L., Zhuang, X., Masten, V. L., Sharp, R. F., & Geyer, M. A. (2009). A Reverse-Translational Study of Dysfunctional Exploration in Psychiatric Disorders: From Mice to Men. *Archives of General Psychiatry*, *66*(10), 1072–1080. https://doi.org/10.1001/ARCHGENPSYCHIATRY.2009.58

Rhee, I., Shin, M., Hong, S., Lee, K., Kim, S. J., & Chong, S. (2011). On the Levy-Walk Nature of Human Mobility. *IEEE/ACM Transactions on Networking*, *19*(3), 630–643. https://doi.org/10.1109/TNET.2011.2120618

Rosenberg, M., Zhang, T., Perona, P., & Meister, M. (2021). Mice in a labyrinth show rapid learning, sudden insight, and efficient exploration. *ELife*, *10*. https://doi.org/10.7554/eLife.66175

Särkelä, H., Takatalo, J., May, P., Laakso, M., & Nyman, G. (2009). The movement patterns and the experiential components of virtual environments. *International Journal of Human Computer Studies*, *67*(9), 787–799. https://doi.org/10.1016/j.ijhcs.2009.05.003

Schomaker, J., Baumann, V., Marit, &, & Ruitenberg, F. L. (2022). Effects of exploring a novel environment on memory across the lifespan. *Scientific Reports 2022 12:1*, *12*(1), 1–13. https://doi.org/10.1038/s41598-022-20562-4

Šimon, M., Vašát, P., Poláková, M., Gibas, P., & Daňková, H. (2019). Activity spaces of homeless men and women measured by GPS tracking data: A comparative analysis of Prague and Pilsen. *Cities*, *86*(August), 145–153. https://doi.org/10.1016/j.cities.2018.09.011

Weaver, W. (1953). Recent contributions to the mathematical theory of communication. *ETC: A Review of General Semantics*, *74*(1), 261–281. http://waste.informatik.hu-berlin.de/Lehre/ss11/SE_Kybernetik/reader/weaver.pdf

Yaremych, H. E., Kistler, W. D., Trivedi, N., & Persky, S. (2019). Path Tortuosity in Virtual Reality: A Novel Approach for Quantifying Behavioral Process in a Food Choice Context. *Cyberpsychology, Behavior, and Social Networking*, *22*(7), 486–493. https://doi.org/10.1089/cyber.2018.0644
